# Supplementary material for: Cranial endocast of the stem lagomorph Megalagus and brain structure of basal Euarchontoglires
Source: Proc Biol Sci. 2020 Jun 24;287(1929):20200665. doi: 10.1098/rspb.2020.0665 (PMC7329053; doi:10.1098/rspb.2020.0665)
Supplement: Supplementary material [file rspb20200665supp1.pdf]

Supporting Material

for Proceedings of the Royal Society B; doi:10.1098/rspb.2020.0665

Cranial endocast of the stem lagomorph *Megalagus* and brain structure of basal Euarchontoglires

Sergi López-Torres[1,2,3,4], Ornella C. Bertrand[5], Madlen M. Lang[6] , Mary T. Silcox[6] and Łucja Fostowicz-Frelik[4,7,8*]

[1]Division of Paleontology, American Museum of Natural History, New York, NY, United States of America
[2]Richard Gilder Graduate School, American Museum of Natural History, New York, NY, United States of America
[3]New York Consortium in Evolutionary Primatology, New York, NY, United States of America
[4]Department of Evolutionary Paleobiology, Institute of Paleobiology, Polish Academy of Sciences, Warsaw, Poland
[5]School of Geosciences, Grant Institute, University of Edinburgh, Edinburgh, EH9 3FE, Scotland, UK
[6]Department of Anthropology, University of Toronto Scarborough, Toronto, ON, Canada
[7]Key Laboratory of Vertebrate Evolution and Human Origins, Institute of Vertebrate Paleontology and Paleoanthropology, Chinese Academy of Sciences, Beijing 100044, China
[8]CAS Center for Excellence in Life and Paleoenvironment, Beijing 100044, China

* Corresponding Author: Łucja Fostowicz-Frelik, Institute of Paleobiology, Polish Academy of Sciences, 51/55 Twarda St, Warsaw 00-818, Poland; email lfost@twarda.pan.pl

Table of contents

Materials and Methods 3

Figures

Fig. S1 5

Fig. S2 6

Fig. S3 7

Fig. S4 8

Fig. S5 9

Fig. S6 10

Fig. S7 11

Fig. S8 12

Fig. S9 13

Tables

Table S1 14

Table S2 15

Table S3 17

Table S4 18

Table S5a 20

Table S5b 24

Table S6 50

Table S7 53

Materials and Methods

The skull of *Megalagus turgidus* (FMNH UC 1642) comes from the early Orellan deposits (early Oligocene; 33.7–32.00 Ma) [24] of the Brule Formation at Grime's Ranch, Sioux County, Nebraska [25]. The specimen is an almost complete (the zygomatic arches are missing), undistorted cranium (Fig. 2), associated with two mandibular bodies, and was originally described by Olson [25].

The skull of *Megalagus turgidus* (Fig. 2) was micro-CT scanned in a high resolution Phoenix v|tome|x L 240 scanner (GE Measurement & Control Solutions) at the American Museum of Natural History (New York, NY, USA) with the following parameters: voltage 155 kV, current 145 mA, and 0.2 mm Cu filter. To accommodate the length of the specimen, the skull was scanned as a multiscan in four parts. The total of 4501 images were acquired at a resolution of 22.54 µm (isotropic voxels) with 0.33 sec of exposure. Raw data were reconstructed with Phoenix datos|x 2.0 software resulting in 16-bit TIFFs (1977x1000 pixel in size). The CT-data of the endocranial cavity of *Megalagus turgidus* were manually segmented in Avizo 9.0.1 (Visualization Science Group, 1995–2015) using a WACOM Cintiq 21UX tablet in each of the three parts that contain the cranial cavity, then the three resulting datasets were merged. We compared the endocast of *Megalagus* to a sample of 10 extant lagomorph species or subspecies representing extant families (Leporidae and Ochotonidae; Figs S3, S4, see also Table S2), and to previously published endocasts of early members of Euarchontoglires (Table S3), including basal Glires (*Rhombomylus*) [5], Palaeogene rodents [35–39], plesiadapiforms [31–34], and the apatemyids *Carcinella* [43] and *Labidolemur* [42].

The comparative material of extant lagomorphs was CT-scanned, with each specimen imaged as a single scan (Table S1), at the Shared Materials Instrumentation Facility (SMIF), Duke University (Durham, NC, USA), apart from *Romerolagus diazi* which was imaged as a multiscan performed at the American Museum of Natural History.

Endocast nomenclature follows Silcox et al. [31], with modifications; in particular, we refer to the paraflocculi as 'petrosal lobules'. Linear measurements are in Fig. S1, surface area and volumetric measurements follow Bertrand and Silcox [36]. They were taken on the endocasts using Avizo 9.0.1 (see Tables 1, S2–S3). Because of poorer preservation on the left side of the *Megalagus* endocast, we followed Jerison [27] and Long et al. [28], who measured only one side of the neocortex (the most complete hemisphere), excluding the superior sagittal sinus, and then doubled the area of the hemisphere (the module 'volume edit' was used). The resulting data on the endocast volume, relative neocortical surface area, relative olfactory bulb and petrosal lobule volumes for *Megalagus* are presented in Table 1. The surface rendering of the *Megalagus* endocast used in this paper is available from the Dryad Digital Repository (doi:10.5061/dryad.0vt4b8gwg) [26].

For comparative purposes, we calculated the encephalization quotient (EQ) using two equations: Jerison's (1973) and Eisenberg's [30]. Values for those equations were calculated for fossil and extant lagomorph endocasts and are included in Tables S2, S3 (and Figs 4, S9). The data for fossil euprimates are based on available virtual endocasts (see [40], Kirk et al. 2014, Ramdarshan and Orliac 2016); for raw data see Tables S5a, b. The width of the occipital

condyles (WOC) was used to estimate the body mass (Moncunill-Solé et al. 2015) for all species of lagomorphs in our sample (Table S7).

The statistical data were analyzed using PAST software ver. 2.17c (Hammer et al. 2001); all results, including Principal Component Analysis (PCA) performed using correlation matrix (Figs 5, S8; Table S4), boxplots (Figs 4, S9; Tables S5a, b), bivariate plots, with accompanying least square regression analysis (Figs 4, S5–S6; Table S6), can be found further in the Supplementary Material. The PCA included nine endocast parameters (Table S4) analyzed for 24 species of extant lagomorphs, fossil rodents, plesiadapiforms, and *Megalagus*.

Additional references

Ø. Hammer, D. A. T. Harper, P. D. Ryan, PAST: Paleontological Statistics software package for education and data analysis. *Palaeontol. Electron.* **4**, 1–9 (2001).

H. J. Jerison, Evolution of the Brain and Intelligence (Academic Press, 1973), 482 p.

E. C. Kirk, P. Daghighi, T. E. Macrini, B.-A. S. Bhullar, T. B. Rowe, Cranial anatomy of the Duchesnean primate *Rooneyia viejaensis*: New insights from high resolution computed tomography. *J. Hum. Evol.* **74**, 82–95 (2014).

B. Moncunill-Solé, J. Quintana, X. Jordana, P. Engelbrektsson, M. Köhler, The weight of fossil leporids and ochotonids: Body mass estimation models for the order Lagomorpha. *J. Zool.* **295**, 269–278 (2015).

A. Ramdarshan, M. J. Orliac, Endocranial morphology of *Microchoerus erinaceus* (Euprimates, Tarsiiformes) and early evolution of the Euprimates brain. *Am. J. Phys. Anthropol.* **159**, 5–16 (2016).

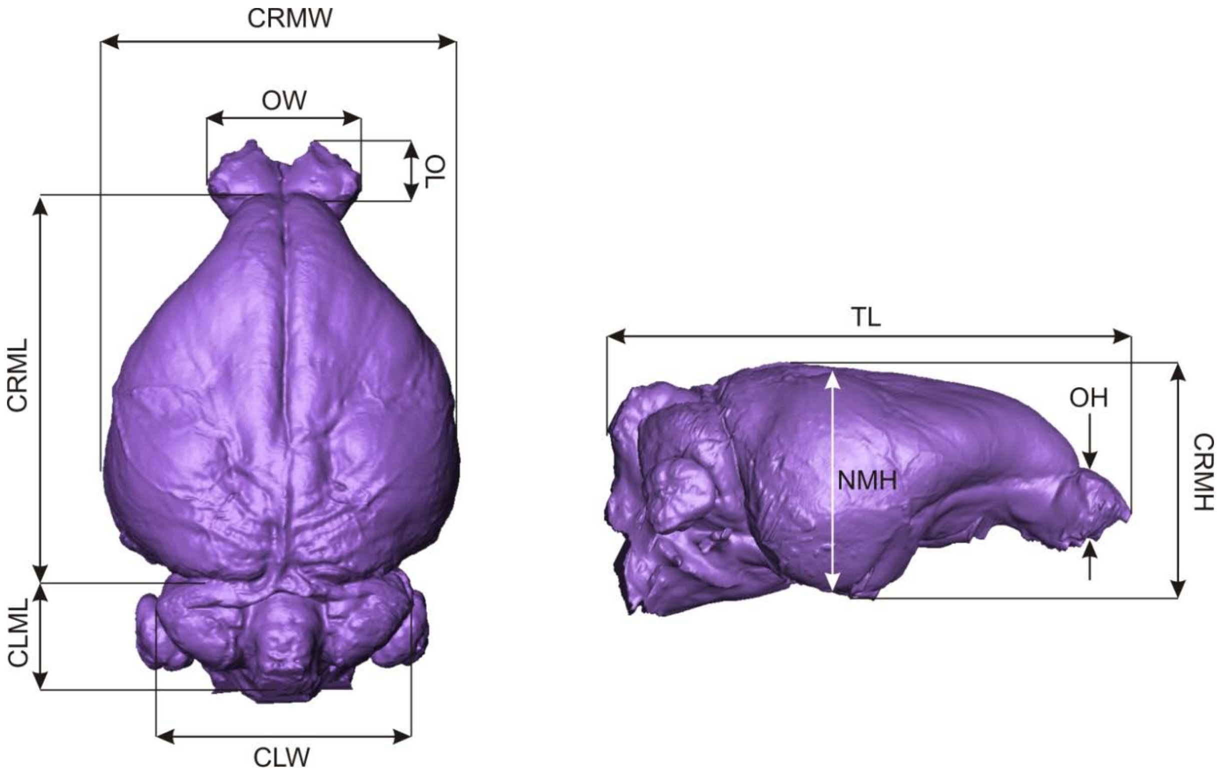

Fig. S1. Linear measurements of endocast (based of *Lepus arcticus* AMNH 42139). Abbreviations: CLML, cerebellum maximum length; CLW, cerebellum width (without petrosal lobes); CRMH, cerebrum maximum height; CRML, cerebrum maximum length; CRMW, cerebrum maximum width; NMH, neocortex maximum height; OH, olfactory bulbs height; OL, olfactory bulbs length; OW, olfactory bulbs width; TL, total endocast length. Left, dorsal; right, lateral view.

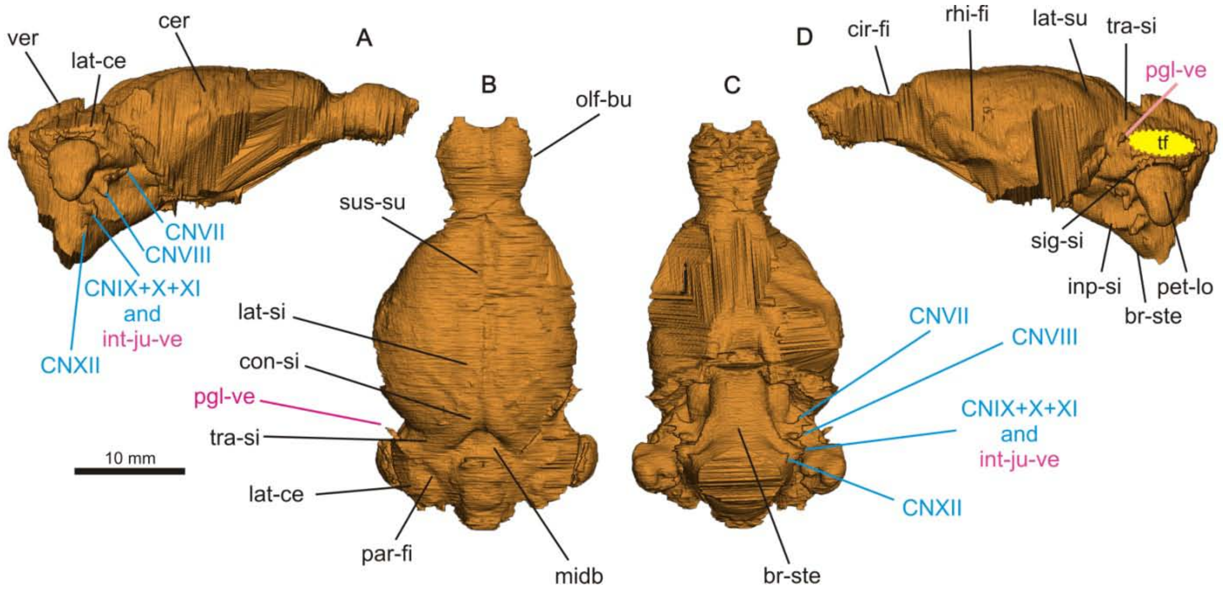

Fig. S2. Digital endocast of *Megalagus turgidus* in (A, D) lateral, (B) dorsal, and (C) ventral views. Abbreviations: br-ste, brain stem; cer, cerebrum; cir-fi, circular fissure; CNVII, cranial nerve VII (facial nerve); CNVIII, cranial nerve VIII (vestibulocochlear nerve); CNIX, cranial nerve IX (glossopharyngeal nerve); CNX, cranial nerve X (vagus nerve); CNXI, cranial nerve XI (accessory nerve); CNXII, cranial nerve XII (hypoglossal nerve); con-si, confluence of sinuses; inp-si, inferior petrosal sinus; int-ju-ve, internal jugular vein; lat-ce, lateral lobe of cerebellum; lat-si, lateral sinus; lat-su, lateral sulcus; midb, midbrain; olf-bu, olfactory bulbs; par-fi, paramedian fissure; pet-lo, petrosal lobule; pgl-ve, postglenoid vein; rhi-fi, rhinal fissure; sig-si, sigmoid sinus; sus-su, superior sagittal sulcus; tf, temporal foramen; tra-si, transverse sinus; ver, vermis. Color code: blue, nerves; pink, blood vessels; black, brain structures.

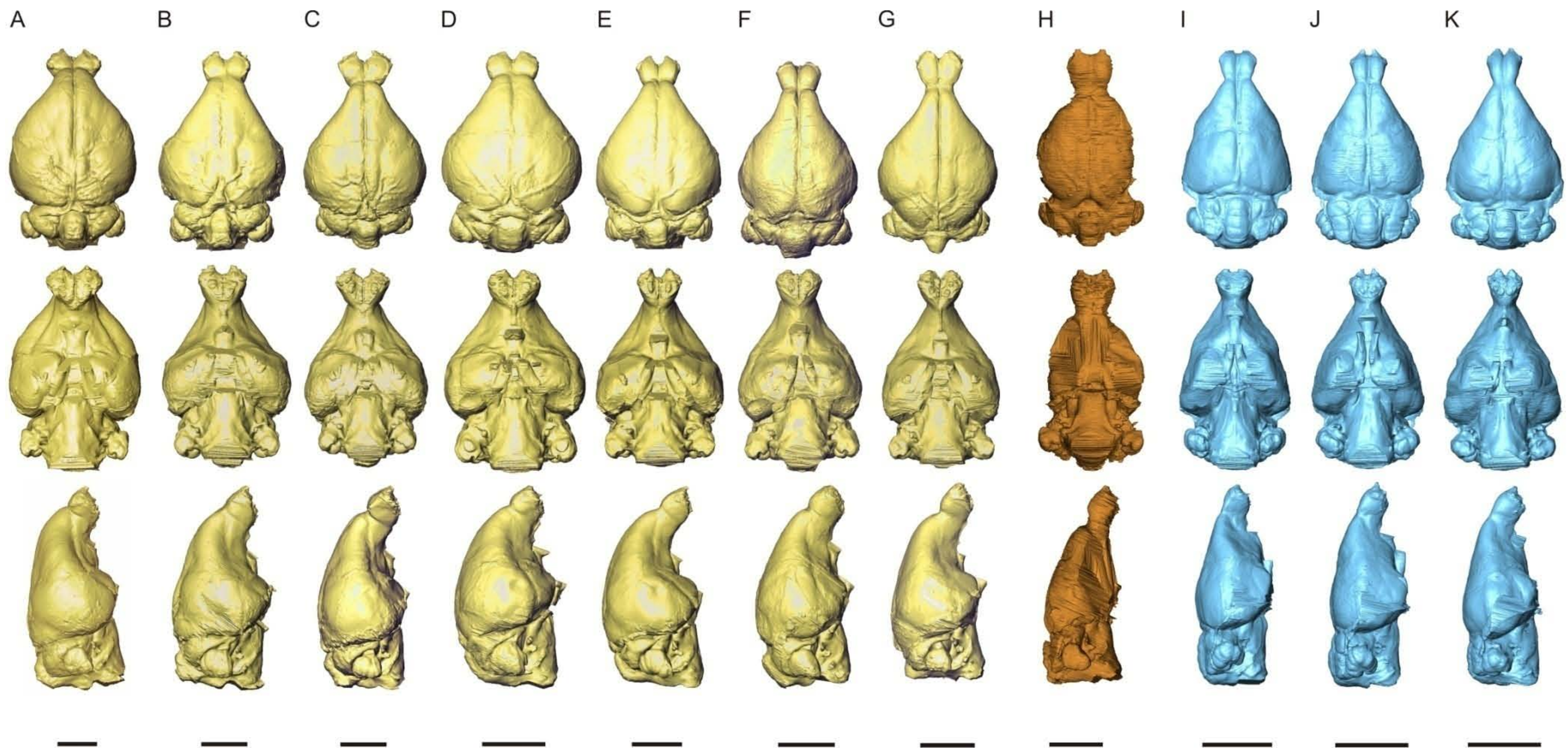

Fig. S3. Comparative endocast morphology of extant Lagomorpha (A–G, I–K) and *Megalagus turgidus* FMNH UC 1642 (H, in orange-brown). Leporidae (in yellow): *Lepus arcticus*, AMNH 42139 (A); *L. americanus bairdii*, AMNH 99352 (B); *L. americanus phaeonotus*, AMNH 99352 (C); *Brachylagus idahoensis*, AMNH 92869 (D); *Oryctolagus cuniculus*, AMNH 34816 (E); *Romerolagus diazi*, AMNH 148172 (F); *Poelagus marjorita*, AMNH 51052 (G). Ochotonidae (in blue): *Ochotona princeps schisticeps*, AMNH 40547 (I); *O. princeps princeps*, AMNH 120698 (J); *O. pallasi*, AMNH 59712 (K). Scale bar represents 1 cm.

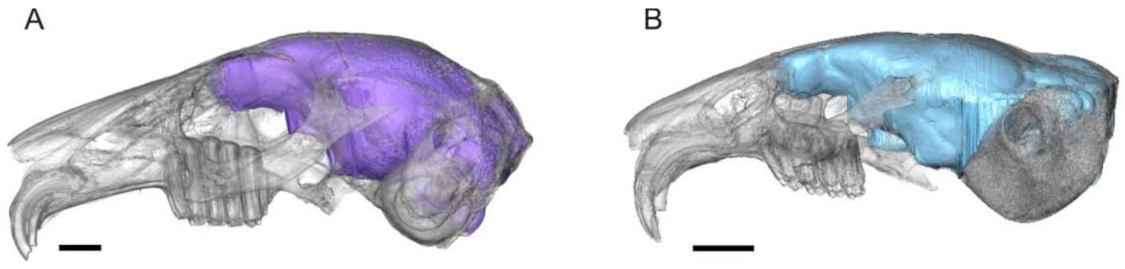

Fig. S4. Lateral view of the extant lagomorph brain endocasts inside translucent crania. A, leporid, *Romerolagus diazi* (AMNH 148172); B, ochotonid, *Ochotona princeps* (AMNH 120698). The picture shows the position of the anterior extremities of the olfactory bulbs in relation to the upper tooth row. Scale bar represents 1 cm.

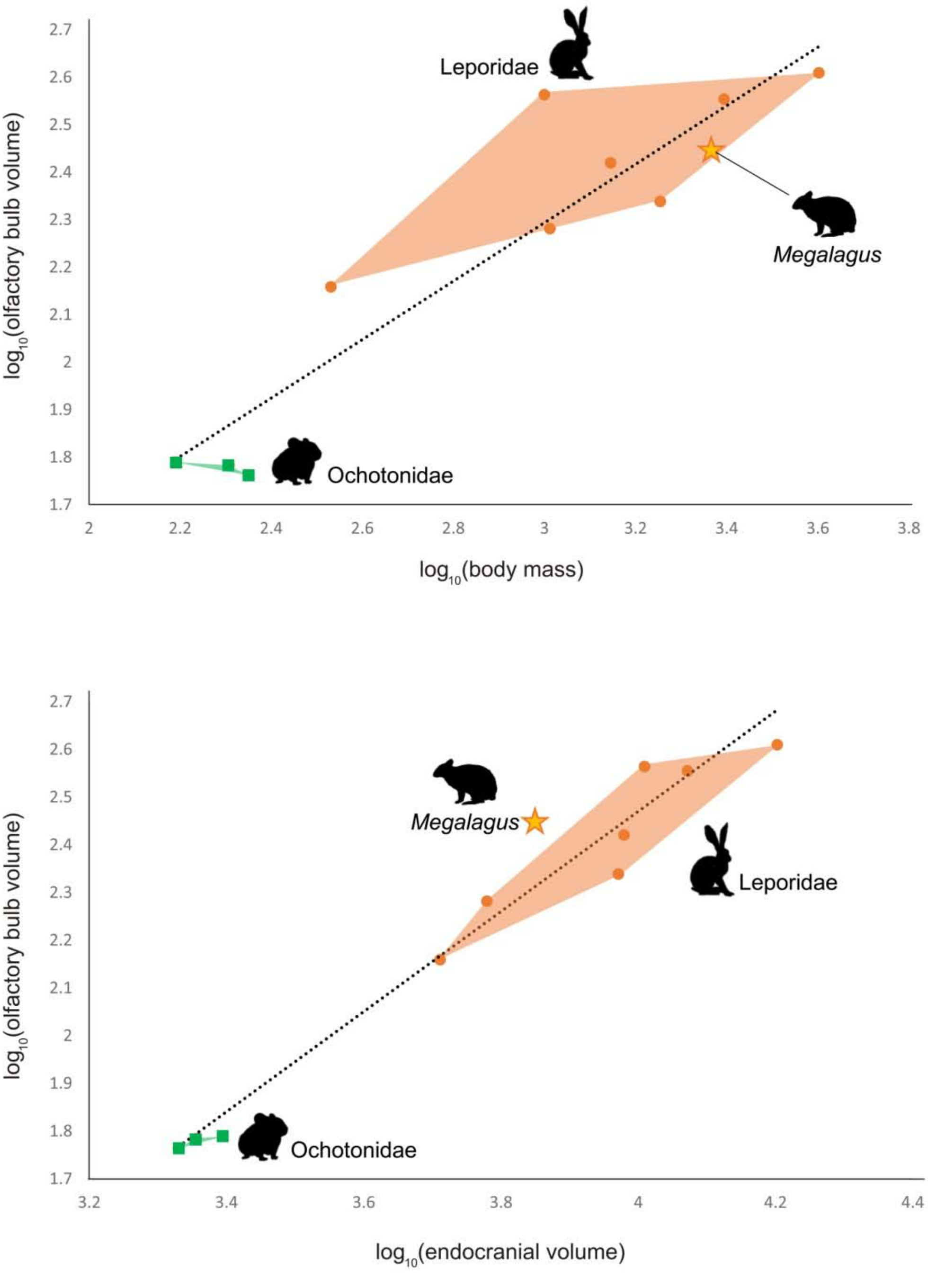

Fig. S5. Olfactory bulb proportions in studied Lagomorpha. Bivariate plots of olfactory bulb volume to body mass (top), and the endocranial volume (down). See metric data in Tables S2, S7. The equation parameters for the least square regression analysis are as follows: slope 0.6154, intercept 0.4475, and r^2 0.8659 for (top), and slope 1.0487, intercept -1.7248, and r^2 0.957 for (down).

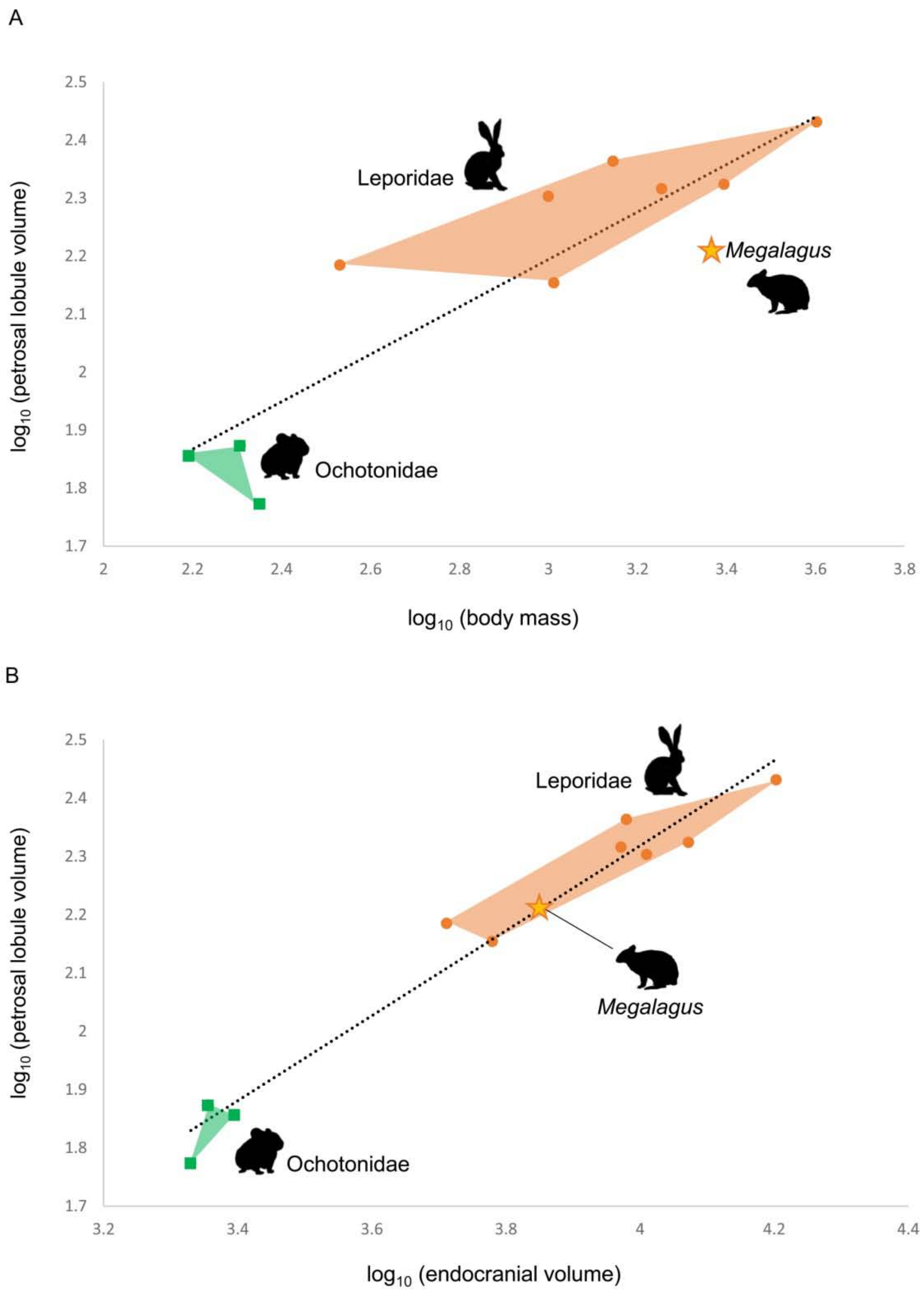

Fig. S6. Petrosal lobule ratios in studied Lagomorpha. Metric data in Tables 1, S2, and S7. The equation parameters for the least square regression analysis are as follows: slope 0.4091, intercept 0.9671, and r^2 0.7977 for (A), and slope 0.7292, intercept -0.5986, and r^2 0.9649 for (B).

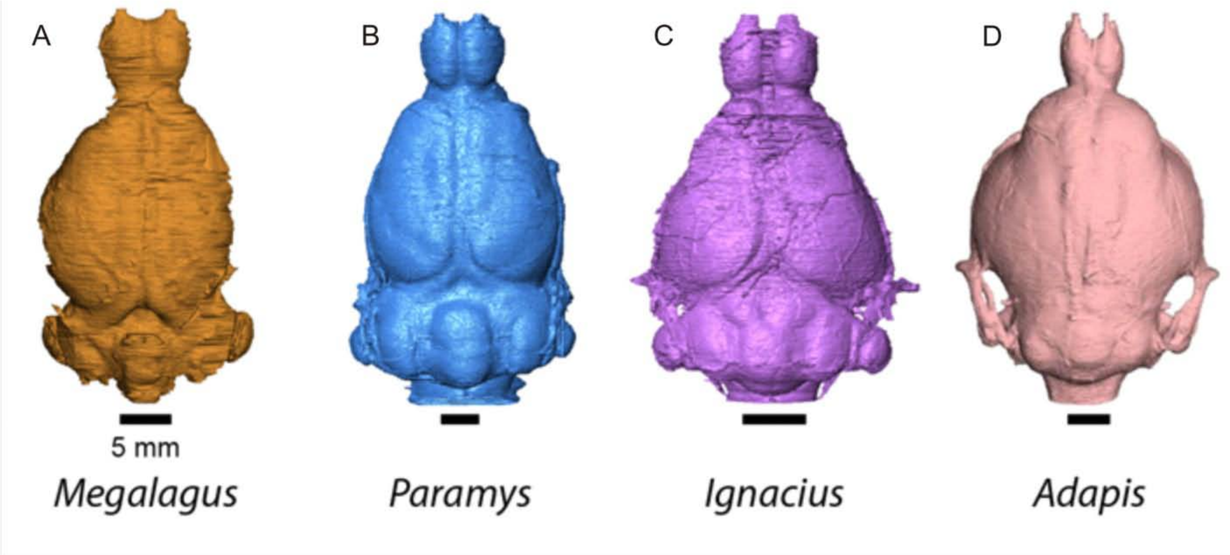

Fig. S7. Comparison of endocast morphology in major groups of Euarchontoglires. A, lagomorph; B, ischyromyid rodent; C, plesiadapiform (stem primate); D, euprimate.

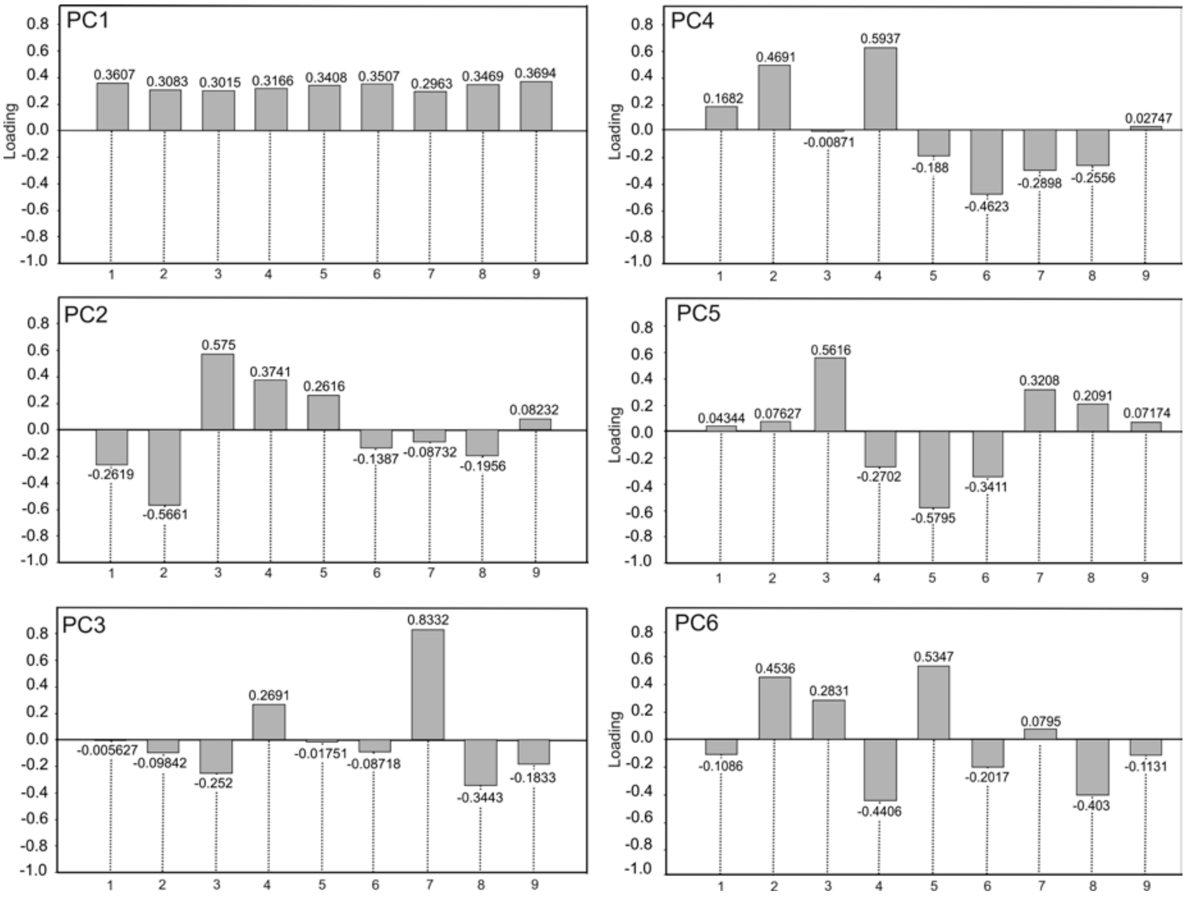

PC	Eigenvalue	% variance
1	6,98839	77,649
2	0,710952	7,8995
3	0,462316	5,1368
4	0,35083	3,8981
5	0,284515	3,1613
6	0,0953711	1,0597
7	0,0680578	0,7562
8	0,0222138	0,24682
9	0,0173513	0,19279

Fig. S8. PCA loadings for the first six principal components and the Eigenvalues for all nine components.

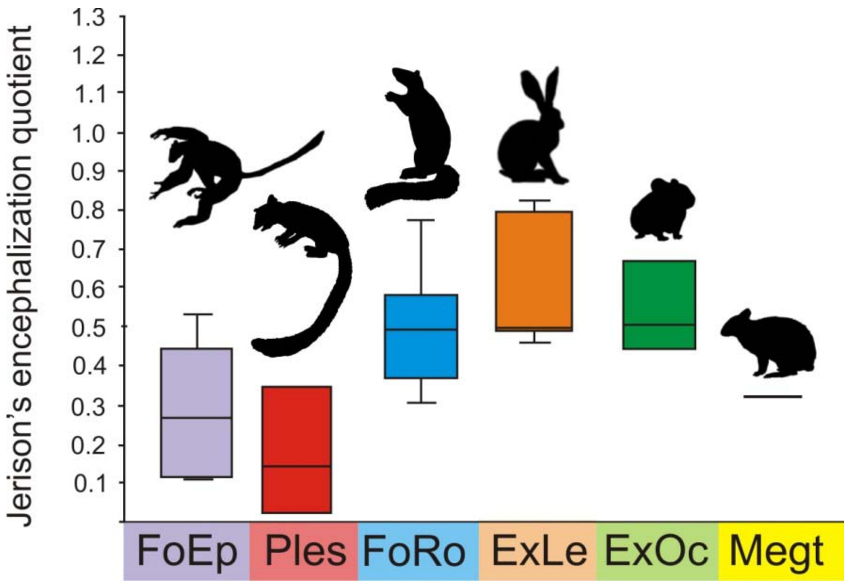

Fig. S9. EQ based on Jerison's equation. Abbreviations: FoEp, fossil eurimates (N=10); Ples, plesiadapids (N=4); FoRo, fossil rodents (N=15); ExLe, extant leporids (N=7); ExOc, extant ochotonids (N=3); Megt, *Megalagus turgidus* (N=1). Metric data in Table S5b.

Table S1. Information on lagomorph CT-scans used in the paper

Species	Specimen	Location of the scan	Author responsible for scanning	Source-object distance (mm)	Energy settings		Number of views	Voxel size (mm)		Columns x rows (total)	Total number of slices
					kV	mA		X and Y	Z		
Brachylagus idahoensis	AMNH 92869	SMIF	MML	118.64	138	87	2000	0.045899	0.091798	1331 x 1393	826
Poelagus marjorita	AMNH 51052	SMIF	MML	194.94	147	91	2000	0.045899	0.091798	1255 x 1316	901
Lepus americanus phaeonotus	AMNH 97648	SMIF	MML	165.24	138	87	2000	0.041922	0.083844	1416 x 1413	988
Lepus americanus bairdii	AMNH 99352	AMNH	OCB	239.11	135	180	2250	0.041191	0.083844	1114 x 1111	926
Lepus arcticus	AMNH 42139	SMIF	MML	233.98	148	90	2000	0.055091	0.082382	1456 x 1384	911
Oryctolagus cuniculus	AMNH 34816	SMIF	MML	158.68	138	87	2000	0.040904	0.110182	1255 x 1394	953.5
Romerolagus diazi	AMNH 148172	AMNH	ŁFF	91.94	150	180	1800	0.022608	0.081808	1669 x 1361	1364
Ochotona princeps princeps	AMNH 120698	SMIF	MML	87.95	129	94	2000	0.023833	0.045216	1255 x 1853	897
Ochotona princeps schisticeps	AMNH 40547	AMNH	OCB	134.26	105	165	2350	0.023128	0.046256	1583 x 1570	998
Ochotona pallasi	AMNH 59712	SMIF	MML	89.27	123	98	2000	0.024188	0.048376	1033 x 1077	906
Megalagus turgidus	FMNH UC 1642	AMNH	ŁFF	91.68	155	145	2250	0.022544	0.022544	1564 x 1421	2701

Table S2. Quantitative endocast data for Lagomorpha

**Measurement**	*Brachylagus idahoensis* AMNH 92869	*Lepus americanus phaeonotus* AMNH 97648	*Lepus arcticus* AMNH 42139	*Lepus americanus bairdii* AMNH 99352	*Oryctolagus cuniculus* AMNH 34816	*Poelagus marjorita* AMNH 51052	*Romerolagus diazi* AMNH 148172	*Ochotona pallasi* AMNH 59712	*Ochotona princeps princeps* AMNH 120698	*Ochotona princeps schisticeps* AMNH 40547
**Linear measurements** (in mm)										
Total length (TL)	33.02	46.36	50.01	43.92	42.70	51.99	38.57	29.73	30.33	31.05
Olfactory bulb length (OL)	5.55	8.6	8.71	10.08	6.98	14.33	6.10	5.34	5.34	4.93
Olfactory bulb width (OW)	8.98	13.68	15.01	10.55	10.09	11.55	9.00	5.39	5.79	6.06
Olfactory bulb height (OH)	6.08	7.82	8.34	7.53	6.58	7.92	6.31	5.03	4.40	4.49
Maximum neocortex height (NMH)	13.44	18.17	17.79	16.13	19.34	15.74	12.73	9.40	9.11	9.78
Cerebrum total length (CRML)	24.94	33.07	35.10	29.15	30.20	34.66	26.72	17.43	16.73	17.94
Cerebrum maximum width (CRMW)	23.00	29.02	32.64	28.02	27.22	29.01	23.49	16.37	16.43	16.81
Cerebrum maximum height (CRMH)	16.38	19.70	22.44	20.83	20.76	20.36	16.85	11.19	11.24	11.39
Cerebellum length (vermis) (CLML)	8.21	11.41	12.06	12.15	8.94	11.23	9.51	6.88	8.30	8.24
Cerebellum width (without paraflocculi) (CLW)	17.34	21.37	23.40	20.83	22.21	21.76	19.14	11.77	12.43	12.13
**Ratios** (in %)										
OL/TL	16.81	18.55	17.42	22.95	16.35	27.56	15.82	17.96	17.61	15.88
CRML/TL	75.53	71.33	70.19	66.37	70.73	66.67	69.28	58.63	55.16	57.78
CLML/TL	24.86	24.61	24.12	27.66	20.94	21.60	24.66	23.14	27.37	26.54
CLW/CRMW	75.39	73.64	71.69	74.34	81.59	75.01	81.48	71.90	75.65	72.16
OW/CRMW	39.04	47.14	45.99	37.65	37.07	39.81	38.31	32.93	35.24	36.05
OW/CLW	51.79	64.01	64.15	50.65	45.43	53.08	47.02	45.79	46.58	49.96
NMH/CRMH	82.05	92.23	79.28	77.44	93.16	77.31	75.55	84.00	81.05	85.86

Table S2. continued

**Measurement**	*Brachylagus idahoensis* AMNH 92869	*Lepus americanus phaeonotus* AMNH 97648	*Lepus arcticus* AMNH 42139	*Lepus americanus bairdii* AMNH 99352	*Oryctolagus cuniculus* AMNH 34816	*Poelagus marjorita* AMNH 51052	*Romerolagus diazi* AMNH 148172	*Ochotona pallasi* AMNH 59712	*Ochotona princeps princeps* AMNH 120698	*Ochotona princeps schisticeps* AMNH 40547
**Surfaces** (in mm^2)										
Total endocast area (TS)	2210.12	3536.03	4562.86	3256.85	3329.57	3778.56	2472.52	1223.86	1270.50	1303.88
Neocortical surface area (NS)	728.43	1255.52	1944.27	1074.20	1223.30	1298.47	720.10	430.58	400.12	433.24
Neocortical surface area (one side) (NS1)	353.76	620.70	957.75	518.48	586.10	627.89	347.10	206.26	196.74	207.56
**Volumes** (in mm^3)										
Total endocast (TV)	5145.19	10221.40	15949.90	9538.09	9363.12	11807.96	6020.62	2138.24	2270.48	2479.85
Olfactory bulbs (OV)	144.28	366.15	407.14	263.19	218.06	358.52	191.21	57.92	60.75	61.63
Petrosal lobules (PLV)	153.04	201.00	270.10	231.02	207.03	210.83	142.54	59.32	74.74	71.80
**Ratio**(in %)										
NS/TS	32.96	35.51	42.61	32.98	36.74	34.36	29.12	35.18	31.49	33.23
OV/TV	2.80	3.58	2.55	2.76	2.33	3.04	3.18	2.71	2.68	2.49
PLV/TV	2.97	1.97	1.69	2.42	2.21	1.79	2.37	2.77	3.29	2.90
**Mass** (in mg)										
Olfactory bulb mass	137.41	348.71	387.75	250.66	207.68	341.45	182.10	55.16	57.86	58.70
Petrosal lobule mass	145.75	191.43	257.24	220.02	197.17	200.79	135.75	56.50	71.18	68.38
Brain volume converted to mass	5.15	1.02	15.95	9.56	9.36	11.81	6.02	2.14	2.27	2.48
**Encephalization quotient**										
**Jerison's EQ**	0.82	0.79	0.49	0.59	0.49	0.50	0.46	0.45	0.51	0.67
**Eisenberg's EQ**	1.19	1.06	0.59	0.78	0.63	0.63	0.61	0.67	0.77	1.02

Table S3. Comparative endocast parameters and EQ values for fossil Euarchontoglires taxa

	Specimen number	Epoch	Total endocast volume (mm^3)	Neocortical surface area ratio (%)	Olfactory bulb volume ratio (%)	Petrosal lobule volume ratio (%)	Jerison's EQ	Eisenberg's EQ
**Rodentia**								
Altasciurus relictus	USNM 437793	Early Oligocene	957.45	-	3.55	3.35	0.78	1.33
Protosciurus cf. *rachelae*	YPM 14736; YPM 14737	Late Oligocene–early Miocene	4546.82–5658.95	30.67–31.71	3.65–4.76	2.96–3.31	0.71	1.03
Paramys copei	AMNH 4756	Early Eocene	7526.65	18.14	6.05	1.20	0.57	0.76
Paramys delicatus	AMNH 12506		12565.40	17.19	4.74	1.03	0.48	0.59
Pseudotomus horribilis	USNM 17159	Middle Eocene	15188.20	18.75	5.33	1.14	0.31	0.36
Pseudotomus oweni	USNM 17161	Middle Eocene	12063.00	21.89	5.94	0.62	0.30	0.36
Pseudotomus petersoni	AMNH 2018	Middle Eocene	17014.90	-	4.14	0.39	0.37	0.43
Pseudotomus hians	AMNH 5025	Middle Eocene	13679.10	23.29	5.43	1.04	0.49	0.61
Rapamys atramontis	AMNH 128706; AMNH 128704	Middle Eocene	6006.47–7109.97	22.98–23.01	3.16–3.76	1.52–2.05	0.46–0.49	0.61–0.66
Ischyromys typus	ROMV 1007; AMNH 12252; AMNH F:AM 144638	Early Oligocene	5578.07–7276.91	19.83–23.41	3.15–3.68	1.60–1.63	0.36–0.53	0.47–0.70
Cedromus wilsoni	USNM 256584	Early Oligocene	3609.87	31.49	2.96	3.16	0.68	0.99
**Primates**								
Microsyops annectens	UW 12362	Middle Eocene	5900.00	24.3	5.1	-	0.32	0.42
Ignacius graybullianus	USNM 421608; UF 26000	Early Eocene	2140.00	21.8–24.4	5.5	-	0.42	0.61
Plesiadapis tricuspidens	MNHN CR 125	Late Palaeocene	5210.00	22	4.9	-	0.12	0.14
Plesiadapis cookei	UM 87990	Late Palaeocene	5000.00	-	7.8	-	0.23	0.29
**Apatemyoidea**								
Labidolemur kayi	USNM 530208; USNM 530221	Late Palaeocene	501.88	-	14.75	-	0.22	0.36

Table S4. Data for Principal Component Analysis of endocast measurements (see Fig. S1 for explanation) for studied lagomorphs, fossil rodents, and plesiadapiforms; var., variable

Measurement<br>Species	TL<br>(var. 1)	OL<br>(var. 2)	OW<br>(var. 3)	CRML<br>(var. 4)	CRMW<br>(var. 5)	CLW<br>(var. 6)	CLML<br>(var. 7)	OV<br>(var. 8)	TV<br>(var. 9)
Plesiadapis tricuspidens (MNHN CR125)	43.5	9.7	4.6	18.2	22	19.6	7.5	136	5210
Plesiadapis cookei (UM 87990)	42	10	5	22	22	20	7.2	390	5700
Microsyops annectens (UW 12362)	41.25	8	5	22.26	24	23.9	11.9	300	5900
Ignacius graybullianus (USNM 421608)	30.79	6.28	3.94	15.8	19.44	15.6	9.4	120	2140
Mesogaulus paniensis (AMNH F:AM 65511)	28.29	4.83	8.11	16.42	21.52	16.15	5.56	114	3468
Protosciurus cf. *rachelae* (YPM 14736)	32.95	6.69	8.35	19.23	22.79	15.81	6.69	216.41	4546.82
Protosciurus cf. *rachelae* (YPM 14737)	36.14	6.73	8.46	19.75	21.8	17.28	6.41	206.7	5658.95
Paramys copei (AMNH 4756)	45.82	10.11	9.63	21.05	21.47	20.3	9.92	455.45	7526.65
Paramys delicatus (AMNH 12506)	50.54	10.17	11.15	23.27	25.63	24.01	11.5	595.51	12565.4
Pseudotomus horribilis (USNM 17159)	54.38	11.76	16.49	26.42	32.11	29.31	12.63	808.92	15188,2
Pseudotomus oweni (USNM 17161)	51.72	10.2	12.49	28.2	23	23.83	10.3	717.06	12063
Pseudotomus hians (AMNH 5025)	47.78	7.82	15.82	23.83	32.34	29.64	12.22	743.2	13679.1
Rapamys atramontis (AMNH 128706)	41.49	7.63	7.99	21.58	22.17	18.66	8.2	224.618	7109.97
Rapamys atramontis (AMNH 128704)	39.48	7.71	8.17	20	22.24	18.34	7.87	226.058	6006.47
Ischyromys typus (ROMV 1007)	40.55	7.24	7.12	20.96	23.58	19.98	9.36	180.09	5578.07
Ischyromys typus (AMNH F:AM 144638)	40.43	7.14	7.73	20.43	23.72	21.44	11.22	229.19	7276.91
Cedromus wilsoni (USNM 256584)	31.98	5.7	6.17	18.61	19.54	12.94	7.03	106.97	3609.87
Megalagus turgidus (FMNH UC 1642)	37.76	8.32	9.6	19.2	18.24	16.32	9.28	280.1	7052.78

Brachylagus idahoensis (AMNH 92869)	33.02	5.55	8.98	24.94	23	17.34	8.21	144.28	5145.19
Lepus americanus (AMNH 97648)	46.36	8.6	13.68	33.07	29.02	21.37	11.41	366.15	10221.4
Lepus americanus bairdii (AMNH 99352)	43.92	10.08	10.55	29.15	28.02	20.83	12.15	263.19	9538.09
Lepus arcticus (AMNH 42139)	50.01	8.71	15.01	35.1	32.64	23.4	12.06	407.14	15949.9
Oryctolagus cuniculus (AMNH 34816)	42.7	6.98	10.09	30.2	27.22	22.21	8.94	218.06	9363.12
Poelagus marjorita (AMNH 51052)	52	14.33	11.55	34.66	29.01	21.76	11.23	358.52	11807.96
Romerolagus diazi (AMNH 148172)	38.57	6.1	9	26.72	23.49	19.14	9.51	191.21	6020.62
Ochotona pallasi (AMNH 59712)	29.73	5.34	5.39	17.43	16.37	11.77	6.88	57.92	2138.24
Ochotona princeps (AMNH 120698)	30.33	5.34	5.79	16.73	16.43	12.43	8.3	60.75	2270,48
Ochotona princeps schisticeps (AMNH 40547)	31.05	4.93	6.06	17.94	16.81	12.13	8.24	61.63	2479.85

Table S5a. Data used for the box plot analyses in Figure 4. NS1*2/TS, neocortical ratio using the neocortical surface area of one side x 2; OV/TV, olfactory bulb volume ratio; PLV/TV, petrosal lobule volume ratio

Group	Species	Collection number	NS1*2/TS	OV/TV	PLV/TV	Source
Extinct Euprimates	*Notharctus tenebrosus*	AMNH 127167	28.89	2.1	-	Harrington et al (2016)
Extinct Euprimates	*Notharctus tenebrosus*	USNM V 23277	31.49	2.23	-	Harrington et al (2016)
Extinct Euprimates	*Notharctus tenebrosus*	USNM V 23278	31.20	1.51	-	Harrington et al (2016)
Extinct Euprimates	*Smilodectes gracilis*	USNM V 17994	30.57	2.06	-	Harrington et al (2016)
Extinct Euprimates	*Smilodectes gracilis*	USNM V 17996	31.21	1.67	-	Harrington et al (2016)
Extinct Euprimates	*Smilodectes gracilis*	USNM V 21815	32.58	1.24	-	Harrington et al (2016)
Extinct Euprimates	*Adapis parisiensis*	NHM M 1345	31.10	2.4	-	Harrington et al (2016)
Extinct Euprimates	*Rooneyia viejaensis*	TMM 40688-7	-	0.94	-	Kirk et al (2014)
Extinct Euprimates	*Microchoerus erinaceus*	UM-PRR 1771	-	0.96	-	Ramdarshan & Orliac (2016)
Plesiadapiformes	*Microsyops annectens*	UW 12362	21.3	5.09	-	Silcox et al (2010a)
Plesiadapiformes	*Ignacius graybullianus*	USNM 421608	19.72	5.53	-	Silcox et al (2009)
Plesiadapiformes	*Plesiadapis cookei*	UM 87990	-	7.8	-	Gingerich & Gunnell (2005)
Plesiadapiformes	*Plesiadapis tricuspidens*	MNHN CR 125	19.9	4.9	-	Orliac et al (2014)
Extinct Rodentia	*Prosciurus relictus*	USNM 437793	-	3.55	3.35	Bertrand et al (2018)
Extinct Rodentia	*Protosciurus* cf. *rachelae*	YPM 14736	30.59	4.76	3.31	Bertrand et al (2018)

Extinct Rodentia	*Protosciurus* cf. *rachelae*	YPM 14737	30.95	3.65	2.96	Bertrand et al (2018)
Extinct Rodentia	*Paramys copei*	AMNH 4756	17.10	6.05	1.20	Bertrand et al (2016b)
Extinct Rodentia	*Paramys delicatus*	AMNH 12506	16.25	4.74	1.03	Bertrand et al (2016b)
Extinct Rodentia	*Pseudotomus horribilis*	USNM 17159	18.89	5.33	1.14	Bertrand et al (2019)
Extinct Rodentia	*Pseudotomus oweni*	USNM 17161	22.92	5.94	0.62	Bertrand et al (2019)
Extinct Rodentia	*Pseudotomus petersoni*	AMNH 2018	22.89	4.14	0.39	Bertrand et al (2019)
Extinct Rodentia	*Pseudotomus hians*	AMNH 5025	23.02	5.43	1.04	Bertrand et al (2019)
Extinct Rodentia	*Rapamys atramontis*	AMNH 128706	20.24	3.16	1.52	Bertrand et al (2019)
Extinct Rodentia	*Rapamys atramontis*	AMNH 128704	21.77	3.76	2.05	Bertrand et al (2019)
Extinct Rodentia	*Ischyromys typus*	ROMV 1007	21.18	3.23	1.63	Bertrand and Silcox, 2016)
Extinct Rodentia	*Ischyromys typus*	AMNH 12252	18.45	3.68	-	Bertrand and Silcox, 2016)
Extinct Rodentia	*Ischyromys typus*	AMNH F:AM 144638	23.03	3.15	1.60	Bertrand and Silcox, 2016)
Extinct Rodentia	*Cedromus wilsoni*	USNM 256584	29.59	2.96	3.16	Bertrand et al (2017)
Extant Rodentia	*Aplodontia rufa*	AMNH 42389	25.94	2.58	0.82	Bertrand et al (2018)
Extant Rodentia	*Sciurus carolinensis*	AMNH 42389	35.41	3.18	2.03	Bertrand and Silcox, 2016)
Extant Rodentia	*Sciurus granatensis*	AMNH 42389	35.39	2.69	2.08	Bertrand et al (2017)
Extant Rodentia	*Tamiasciurus hudsonicus*	USNM 549146	36.30	2.50	2.33	Bertrand et al (2017)
Extant Rodentia	*Eutamias minimus*	USNM 298500	34.93	3.36	2.29	Bertrand et al (2017)
Extant Rodentia	*Funisciurus pyrropus*	USNM 294865	36.44	3.22	2.19	Bertrand et al (2017)

Extant Rodentia	*Heliosciurus rufobrachium*	USNM 378091	35.85	2.45	1.87	Bertrand et al (2017)
Extant Rodentia	*Paraxerus cepapi*	USNM 367956	35.62	2.62	1.46	Bertrand et al (2017)
Extant Rodentia	*Protoxerus stangeri*	USNM 435027	38.02	2.31	1.89	Bertrand et al (2017)
Extant Rodentia	*Aeromys tephromelas*	USNM 481190	35.19	2.85	1.45	Bertrand et al (2017)
Extant Rodentia	*Glaucomys volans*	AMNH 240290	35.69	3.49	1.68	Bertrand et al (2017)
Extant Rodentia	*Petaurista petaurista*	USNM 589079	35.28	1.64	1.62	Bertrand et al (2017)
Extant Rodentia	*Hylopetes spadiceus*	USNM 488639	36.73	3.30	0.85	Bertrand et al (2017)
Extant Rodentia	*Petinomys setosus*	USNM 488674	35.76	3.10	-	Bertrand et al (2017)
Extant Rodentia	*Pteromyscus pulverulentus*	USNM 481178	33.82	2.81	1.37	Bertrand et al (2017)
Extant Rodentia	*Pteromys buechneri*	USNM 172622	34.11	1.75	1.61	Bertrand et al (2017)
Extant Rodentia	*Rhinosciurus laticaudatus*	USNM 488511	34.10	3.88	2.22	Bertrand et al (2017)
Extant Rodentia	*Callosciurus* sp.	USNM 294865	38.79	3.28	1.77	Bertrand et al (2017)
Extant Rodentia	*Lariscus insignis*	USNM 488570	33.88	4.73	2.32	Bertrand et al (2017)
Extant Rodentia	*Dremomys rufigenis*	USNM 488602	36.82	3.96	2.14	Bertrand et al (2017)
Extant Rodentia	*Ratufa affinis*	USNM 488104	37.09	1.64	1.91	Bertrand et al (2017)
Extant Leporidae	*Brachylagus idahoensis*	AMNH 92869	32.01	2.80	2.97	This paper
Extant Leporidae	*Lepus americanus bairdii*	AMNH 42139	31.84	2.76	2.42	This paper
Extant Leporidae	*Lepus americanus phaeonotus*	AMNH 51052	35.11	3.58	1.97	This paper

Extant Leporidae	*Lepus arcticus*	AMNH 97648	41.98	2.55	1.69	This paper
Extant Leporidae	*Oryctolagus cuniculus*	AMNH 99352	35.21	2.33	2.21	This paper
Extant Leporidae	*Poelagus marjorita*	AMNH 34816	33.23	3.04	1.79	This paper
Extant Leporidae	*Romerolagus diazi*	AMNH 148172	28.08	3.18	2.37	This paper
Extant Ochotonidae	*Ochotona pallasi*	AMNH 120698	33.71	2.71	2.77	This paper
Extant Ochotonidae	*Ochotona princeps princeps*	AMNH 59712	30.97	2.68	3.29	This paper
Extant Ochotonidae	*Ochotona princeps schisticeps*	AMNH 40547	31.84	2.49	2.90	This paper
Stem lagomorph	*Megalagus turgidus*	FMNH UC 1642	18.98	3.97	2.31	This paper
Apatemyid	*Labidolemur kayi*	USNM 530208/530221	-	13.02	-	Silcox et al (2011)

Table S5b. Data used for the box plot analyses in Figure 4 and S9. EQ, encephalization quotient

Group	Species	Body mass (g)	Brain mass (g)	Jerison's EQ	Eisenberg's EQ	Source
Extant Leporidae	*Brachylagus idahoensis*	339.56	4.90	0.82835	1.18741999	This paper
Extant Leporidae	*Lepus americanus bairdii*	1396.22	9.11	0.593178	0.7753435	This paper
Extant Leporidae	*Lepus americanus phaeonotus*	998.68	9.73	0.793465	1.06175485	This paper
Extant Leporidae	*Lepus arcticus*	4003.10	15.19	0.488425	0.59304291	This paper
Extant Leporidae	*Oryctolagus cuniculus*	1796.07	8.917	0.490517	0.62995199	This paper
Extant Leporidae	*Romerolagus diazi*	1027.79	5.73	0.458422	0.61219303	This paper
Extant Leporidae	*Poelagus marjorita*	2480.24	11.25	0.4983	0.6256518	This paper
Extant Ochotonidae	*Ochotona pallasi*	223.85	2.04	0.452043	0.67164444	This paper
Extant Ochotonidae	*Ochotona princeps princeps*	202.92	2.16	0.512629	0.76691336	This paper
Extant Ochotonidae	*Ochotona princeps schisticeps*	155.10	2.36	0.670416	1.02201659	This paper
Stem lagomorph	*Megalagus turgidus*	2325.01	6.72	0.310803	0.39200494	This paper
Extinct Rodentia	*Prosciurus relictus*	30.07	0.91	0.776951	1.32855846	Bertrand et al (2018)
Extinct Rodentia	*Protosciurus* cf. *rachelae*	349.62	4.33	0.713063	1.02690931	Bertrand et al (2018)
Extinct Rodentia	*Paramys copei*	1029.89	7.17	0.57235	0.76422646	Bertrand et al (2016b)
Extinct Rodentia	*Paramys delicatus*	2913.82	11.97	0.47601	0.59096189	Bertrand et al (2016b)
Extinct Rodentia	*Pseudotomus horribilis*	7466.70	14.47	0.306295	0.35602181	Bertrand et al (2019)
Extinct Rodentia	*Pseudotomus oweni*	5396.00	11.49	0.30241	0.3595902	Bertrand et al (2019)

Extinct Rodentia	*Pseudotomus petersoni*	6644.56	16.20	0.371028	0.43480043	Bertrand et al (2019)
Extinct Rodentia	*Pseudotomus hians*	3153.50	13.03	0.491469	0.60678718	Bertrand et al (2019)
Extinct Rodentia	*Rapamys atramontis*	1307.61	6.77	0.460741	0.605006	Bertrand et al (2019)
Extinct Rodentia	*Rapamys atramontis*	918.93	5.72	0.493003	0.66355341	Bertrand et al (2019)
Extinct Rodentia	*Ischyromys typus*	1342.23	5.31	0.355197	0.46556207	Bertrand and Silcox, 2016)
Extinct Rodentia	*Ischyromys typus*	1086.42	5.65	0.43541	0.57920863	Bertrand and Silcox, 2016)
Extinct Rodentia	*Ischyromys typus*	1109.01	6.93	0.526586	0.6994881	Bertrand and Silcox, 2016)
Extinct Rodentia	*Cedromus wilsoni*	268.89	3.44	0.675003	0.99012925	Bertrand et al (2017)
Plesiadapiformes	*Microsyops annectens*	1686	5.62	0.322468	0.41597093	Silcox et al (2010a)
Plesiadapiformes	*Ignacius graybullianus*	253	2.04	0.416824	0.6140308	Silcox et al (2009)
Plesiadapiformes	*Plesiadapis tricuspidens*	6372	4.96	0.116843	0.13732804	Orliac et al (2014)
Plesiadapiformes	*Plesiadapis cookei*	2200	4.76	0.228653	0.28950948	Gingerich & Gunnell (2005)
Apatemyidae	*Labidolemur kayi*	74	0.478	0.222762	0.35764366	Silcox et al (2011)
Extinct Euprimates	*Notharctus tenebrosus*	2641	7.03	0.298608	0.37327879	Harrington et al (2016)
Extinct Euprimates	*Notharctus tenebrosus*	2923	7.68	0.304691	0.3781876	Harrington et al (2016)
Extinct Euprimates	*Notharctus tenebrosus*	2244	7.08	0.3353	0.42395277	Harrington et al (2016)
Extinct Euprimates	*Smilodectes gracilis*	1420	8.22	0.529184	0.69088059	Harrington et al (2016)
Extinct Euprimates	*Smilodectes gracilis*	1303	8.56	0.583951	0.76698416	Harrington et al (2016)
Extinct Euprimates	*Smilodectes gracilis*	1582	7.09	0.42436	0.54985188	Harrington et al (2016)
Extinct Euprimates	*Smilodectes gracilis*	1547	8.019	0.487509	0.63266649	Harrington et al (2016)

Extinct Euprimates	*Adapis parisiensis*	1074	8.39	0.651377	0.86719808	Harrington et al (2016)
Extinct Euprimates	*Rooneyia viejaensis*	381	6.89	1.070997	1.53313215	Kirk et al (2014)
Extinct Euprimates	*Microchoerus erinaceus*	597	4.06	0.466806	0.64755128	Ramdarshan & Orliac (2016)
Extant Rodentia	*Acomys dimidiatus*	76	0.97	0.444064	0.7116141	Bertrand et al (2018)
Extant Rodentia	*Acomys dimidiatus*	50.5	0.88	0.532189	0.87758969	Bertrand et al (2018)
Extant Rodentia	*Aeromys tephromelas*	904.59	10.92	0.95071	1.28101056	Bertrand et al (2018)
Extant Rodentia	*Allactaga elater*	80	1.90	0.840431	1.34196713	Bertrand et al (2018)
Extant Rodentia	*Allactaga sibirica*	193	3.50	0.85815	1.28834004	Bertrand et al (2018)
Extant Rodentia	*Allactaga sibirica*	106	2	0.732645	1.1470399	Bertrand et al (2018)
Extant Rodentia	*Aplodontia rufa*	1475.86	7.52	0.471755	0.61424253	Bertrand et al (2018)
Extant Rodentia	*Aplodontia rufa*	982	7.60	0.626495	0.83931688	Bertrand et al (2018)
Extant Rodentia	*Aplodontia rufa*	870	8.40	0.750966	1.01463516	Bertrand et al (2018)
Extant Rodentia	*Aplodontia rufa*	710	8.40	0.860508	1.17929641	Bertrand et al (2018)
Extant Rodentia	*Aplodontia rufa*	985	8.80	0.723934	0.96964939	Bertrand et al (2018)
Extant Rodentia	*Aplodontia rufa*	887	8.30	0.732467	0.98830156	Bertrand et al (2018)
Extant Rodentia	*Apodemus agrarius*	28.50	0.63	0.5582	0.95809098	Bertrand et al (2018)
Extant Rodentia	*Apodemus agrarius agrarius*	26.20	0.62	0.57936	1.00028465	Bertrand et al (2018)
Extant Rodentia	*Apodemus flavicollis*	30	0.75	0.640042	1.09462762	Bertrand et al (2018)
Extant Rodentia	*Apodemus flavicollis flavicollis*	33	0.80	0.640478	1.0880893	Bertrand et al (2018)
Extant Rodentia	*Apodemus sylvaticus*	19.40	0.59	0.674285	1.18892265	Bertrand et al (2018)

Extant Rodentia	*Apodemus sylvaticus*	22	0.56	0.592481	1.03552643	Bertrand et al (2018)
Extant Rodentia	*Apodemus sylvaticus*	21.60	0.59	0.627461	1.09807273	Bertrand et al (2018)
Extant Rodentia	*Apodemus sylvaticus sylvaticus*	19	0.60	0.695353	1.22785888	Bertrand et al (2018)
Extant Rodentia	*Arvicola amphibius*	137.80	1.53	0.470125	0.7226398	Bertrand et al (2018)
Extant Rodentia	*Arvicola amphibius*	131	1.50	0.476802	0.73550456	Bertrand et al (2018)
Extant Rodentia	*Arvicola amphibius*	84.50	1.09	0.463078	0.73659833	Bertrand et al (2018)
Extant Rodentia	*Atherurus africanus*	1600	17.20	1.02232	1.3235932	Bertrand et al (2018)
Extant Rodentia	*Atherurus africanus*	3620	25.30	0.870171	1.06402308	Bertrand et al (2018)
Extant Rodentia	*Atherurus africanus*	1925	17.80	0.934696	1.19458244	Bertrand et al (2018)
Extant Rodentia	*Atherurus africanus*	2250	23	1.087888	1.37526896	Bertrand et al (2018)
Extant Rodentia	*Atlantoxerus getulus*	251	3.75	0.771515	1.13716435	Bertrand et al (2018)
Extant Rodentia	*Brachytarsomys albicauda*	300	2.47	0.450655	0.65599736	Bertrand et al (2018)
Extant Rodentia	*Brachyuromys ramirohitra*	94	1.40	0.555842	0.87758318	Bertrand et al (2018)
Extant Rodentia	*Callosciurus* sp.	437.35	6.67	0.945942	1.34110378	Bertrand et al (2018)
Extant Rodentia	*Callospermophilus lateralis*	246	2.98	0.621223	0.91693369	Bertrand et al (2018)
Extant Rodentia	*Callospermophilus lateralis*	217	3.10	0.701592	1.0446931	Bertrand et al (2018)
Extant Rodentia	*Capromys pilorides*	7000	11	0.243218	0.28398474	Bertrand et al (2018)
Extant Rodentia	*Castor canadensis*	20500	44	0.473583	0.51289644	Bertrand et al (2018)
Extant Rodentia	*Castor canadensis*	18000	53	0.622389	0.68021951	Bertrand et al (2018)
Extant Rodentia	*Castor canadensis*	14500	45	0.610822	0.67775864	Bertrand et al (2018)

Extant Rodentia	*Castor canadensis*	22500	52	0.525847	0.56580027	Bertrand et al (2018)
Extant Rodentia	*Castor canadensis*	8000	38	0.768302	0.88873416	Bertrand et al (2018)
Extant Rodentia	*Castor canadensis*	9750	48	0.850016	0.96973415	Bertrand et al (2018)
Extant Rodentia	*Castor canadensis*	12500	42	0.629707	0.70600983	Bertrand et al (2018)
Extant Rodentia	*Castor canadensis*	16000	38	0.482883	0.53212012	Bertrand et al (2018)
Extant Rodentia	*Castor canadensis*	5500	43	1.117491	1.32701226	Bertrand et al (2018)
Extant Rodentia	*Castor canadensis*	20000	40	0.437712	0.47486774	Bertrand et al (2018)
Extant Rodentia	*Castor canadensis*	4180	25.48	0.795848	0.96339379	Bertrand et al (2018)
Extant Rodentia	*Castor canadensis*	5380	29.52	0.778594	0.92600288	Bertrand et al (2018)
Extant Rodentia	*Castor canadensis*	14500	45	0.610822	0.67775864	Bertrand et al (2018)
Extant Rodentia	*Castor fiber*	14300	41.40	0.56721	0.62997968	Bertrand et al (2018)
Extant Rodentia	*Castor fiber*	16900	38.80	0.475298	0.52175958	Bertrand et al (2018)
Extant Rodentia	*Castor fiber*	25000	45	0.424045	0.45290986	Bertrand et al (2018)
Extant Rodentia	*Castor fiber*	23100	45.50	0.452075	0.48552702	Bertrand et al (2018)
Extant Rodentia	*Castor fiber*	20000	39	0.426769	0.46299604	Bertrand et al (2018)
Extant Rodentia	*Cavia aperea*	163	2.70	0.741339	1.12621111	Bertrand et al (2018)
Extant Rodentia	*Cavia aperea*	260	3	0.602432	0.88575955	Bertrand et al (2018)
Extant Rodentia	*Cavia aperea*	430	3.90	0.559061	0.7935473	Bertrand et al (2018)
Extant Rodentia	*Cavia aperea*	647	5.46	0.595258	0.82110433	Bertrand et al (2018)
Extant Rodentia	*Cavia aperea*	460	4.20	0.575467	0.81298656	Bertrand et al (2018)

Extant Rodentia	*Cavia aperea*	792	4.70	0.447477	0.60857778	Bertrand et al (2018)
Extant Rodentia	*Cavia aperea*	540	4.10	0.504544	0.70483481	Bertrand et al (2018)
Extant Rodentia	*Cavia aperea* f. *porcellus*	485	4.57	0.604349	0.85063291	Bertrand et al (2018)
Extant Rodentia	*Cavia porcellus*	520	5.40	0.681538	0.95461038	Bertrand et al (2018)
Extant Rodentia	*Cavia porcellus*	650	5.80	0.630369	0.86925456	Bertrand et al (2018)
Extant Rodentia	*Cavia porcellus*	348	6.40	1.05716	1.52295195	Bertrand et al (2018)
Extant Rodentia	*Cavia porcellus*	648	4.40	0.479199	0.66094001	Bertrand et al (2018)
Extant Rodentia	*Cavia porcellus*	493.1	3.80	0.496977	0.69869328	Bertrand et al (2018)
Extant Rodentia	*Cavia porcellus*	361	4.10	0.660804	0.94951842	Bertrand et al (2018)
Extant Rodentia	*Cavia porcellus*	324	3.80	0.658472	0.95335612	Bertrand et al (2018)
Extant Rodentia	*Cavia porcellus*	214.94	3.32	0.757363	1.12849124	Bertrand et al (2018)
Extant Rodentia	*Cavia porcellus*	214.57	3.28	0.749103	1.11631729	Bertrand et al (2018)
Extant Rodentia	*Cavia porcellus*	432	4	0.571616	0.81110464	Bertrand et al (2018)
Extant Rodentia	*Cavia porcellus*	456	4.23	0.582979	0.82410254	Bertrand et al (2018)
Extant Rodentia	*Cavia porcellus*	675	4.54	0.481106	0.66167683	Bertrand et al (2018)
Extant Rodentia	*Cavia porcellus*	971	4.28	0.355489	0.47662455	Bertrand et al (2018)
Extant Rodentia	*Cavia porcellus*	900	4.94	0.431721	0.58191876	Bertrand et al (2018)
Extant Rodentia	*Cavia porcellus*	500	5	0.647857	0.90992809	Bertrand et al (2018)
Extant Rodentia	*Cavia porcellus*	700	4.73	0.489175	0.67106326	Bertrand et al (2018)
Extant Rodentia	*Chaetodipus baileyi*	31.20	0.62	0.514588	0.87765641	Bertrand et al (2018)

Extant Rodentia	*Chaetodipus californicus*	26	0.57	0.536721	0.92716396	Bertrand et al (2018)
Extant Rodentia	*Chaetodipus fallax*	20.30	0.50	0.549048	0.96503229	Bertrand et al (2018)
Extant Rodentia	*Chaetodipus formosus*	15.30	0.41	0.548725	0.98374461	Bertrand et al (2018)
Extant Rodentia	*Chaetodipus hispidus*	35.20	0.63	0.481936	0.81505691	Bertrand et al (2018)
Extant Rodentia	*Chaetodipus penicillatus*	16.50	0.42	0.533787	0.95191929	Bertrand et al (2018)
Extant Rodentia	*Chaetodipus spinatus*	19.10	0.44	0.505935	0.89305603	Bertrand et al (2018)
Extant Rodentia	*Chinchilla chinchilla*	520	7.80	0.984444	1.37888167	Bertrand et al (2018)
Extant Rodentia	*Chinchilla chinchilla*	425	6.40	0.924652	1.31355129	Bertrand et al (2018)
Extant Rodentia	*Chinchilla chinchilla*	320	6.90	1.205639	1.74708074	Bertrand et al (2018)
Extant Rodentia	*Chinchilla chinchilla*	470	8.90	1.201996	1.69555726	Bertrand et al (2018)
Extant Rodentia	*Chinchilla chinchilla*	450	6	0.834291	1.18045343	Bertrand et al (2018)
Extant Rodentia	*Chinchilla lanigera*	432	5.20	0.743101	1.05443604	Bertrand et al (2018)
Extant Rodentia	*Chinchilla lanigera*	380	5.20	0.80978	1.15941349	Bertrand et al (2018)
Extant Rodentia	*Chinchilla lanigera*	385	5.05	0.779563	1.11512951	Bertrand et al (2018)
Extant Rodentia	*Chinchilla lanigera*	460	5.10	0.698781	0.98719796	Bertrand et al (2018)
Extant Rodentia	*Chinchilla lanigera*	385	4.90	0.756408	1.08200685	Bertrand et al (2018)
Extant Rodentia	*Chinchilla lanigera*	370	5.32	0.843403	1.20981015	Bertrand et al (2018)
Extant Rodentia	*Chinchilla lanigera*	385	5.50	0.849029	1.21449748	Bertrand et al (2018)
Extant Rodentia	*Chinchilla lanigera*	425	6.40	0.924652	1.31355129	Bertrand et al (2018)
Extant Rodentia	*Chinchilla lanigera*	500	6	0.777428	1.0919137	Bertrand et al (2018)

Extant Rodentia	*Colomys goslingi*	58.20	1.37	0.75106	1.22626963	Bertrand et al (2018)
Extant Rodentia	*Cricetomys emini*	1000	6.60	0.53748	0.71914887	Bertrand et al (2018)
Extant Rodentia	*Cricetomys emini*	80.50	2.70	1.189322	1.89823371	Bertrand et al (2018)
Extant Rodentia	*Cricetulus griseus*	36	0.67	0.506024	0.85444836	Bertrand et al (2018)
Extant Rodentia	*Cricetulus griseus*	23.18	0.63	0.637019	1.10930398	Bertrand et al (2018)
Extant Rodentia	*Cricetus cricetus*	450	2.85	0.396288	0.56071538	Bertrand et al (2018)
Extant Rodentia	*Cricetus cricetus*	297	2.20	0.404105	0.58865085	Bertrand et al (2018)
Extant Rodentia	*Cuniculus paca*	5635	35.80	0.915383	1.08516654	Bertrand et al (2018)
Extant Rodentia	*Cuniculus paca*	6125	33.50	0.810033	0.95468735	Bertrand et al (2018)
Extant Rodentia	*Cuniculus paca*	3665	33.20	1.132471	1.38356067	Bertrand et al (2018)
Extant Rodentia	*Cuniculus paca*	9000	37.30	0.696923	0.79954745	Bertrand et al (2018)
Extant Rodentia	*Cuniculus paca*	5000	26.10	0.723018	0.86432599	Bertrand et al (2018)
Extant Rodentia	*Cuniculus paca*	3627	21.85	0.750539	0.9176163	Bertrand et al (2018)
Extant Rodentia	*Cuniculus paca*	4559	48	1.414547	1.70197277	Bertrand et al (2018)
Extant Rodentia	*Cynomys ludovicianus*	1200	6.40	0.461262	0.60934145	Bertrand et al (2018)
Extant Rodentia	*Dasymys incomtus*	102.5	1.57	0.58971	0.92543077	Bertrand et al (2018)
Extant Rodentia	*Dasyprocta leporina*	3600	21.60	0.745675	0.91214686	Bertrand et al (2018)
Extant Rodentia	*Dasyprocta leporina*	2684	20	0.840552	1.04955646	Bertrand et al (2018)
Extant Rodentia	*Dasyprocta leporina*	2390	18.40	0.835815	1.05215142	Bertrand et al (2018)
Extant Rodentia	*Dasyprocta leporina*	2004	17	0.868953	1.10743815	Bertrand et al (2018)

Extant Rodentia	*Dasyprocta leporina*	2880	18.50	0.741647	0.92150173	Bertrand et al (2018)
Extant Rodentia	*Dasyprocta leporina*	2350	18	0.826943	1.04221461	Bertrand et al (2018)
Extant Rodentia	*Dasyprocta leporina*	2550	17	0.739411	0.92658242	Bertrand et al (2018)
Extant Rodentia	*Dasyprocta leporina*	3172	18.30	0.687665	0.84867109	Bertrand et al (2018)
Extant Rodentia	*Dasyprocta leporina*	2371	19.80	0.904232	1.13891342	Bertrand et al (2018)
Extant Rodentia	*Dasyprocta leporina*	2400	27	1.223041	1.53915485	Bertrand et al (2018)
Extant Rodentia	*Dasyprocta leporina*	2370	19.80	0.904487	1.13926901	Bertrand et al (2018)
Extant Rodentia	*Dasyprocta mexicana*	1527	17.80	1.091608	1.41792736	Bertrand et al (2018)
Extant Rodentia	*Dasyprocta mexicana*	2300	20	0.932161	1.17659301	Bertrand et al (2018)
Extant Rodentia	*Dasyprocta punctata*	3172	18.34	0.689168	0.8505261	Bertrand et al (2018)
Extant Rodentia	*Dendromus mesomelas*	14	0.51	0.725242	1.30830891	Bertrand et al (2018)
Extant Rodentia	*Desmodillus auricularis*	60	1.12	0.600725	0.97872564	Bertrand et al (2018)
Extant Rodentia	*Dicrostonyx groenlandicus*	52.1	0.90	0.529613	0.87143772	Bertrand et al (2018)
Extant Rodentia	*Dipodomys deserti*	114.7	1.60	0.555944	0.86560134	Bertrand et al (2018)
Extant Rodentia	*Dipodomys heermanni*	71.3	1.38	0.659823	1.06210467	Bertrand et al (2018)
Extant Rodentia	*Dipodomys merriami*	41	1.06	0.731791	1.22447097	Bertrand et al (2018)
Extant Rodentia	*Dipodomys microps*	65.7	1.12	0.567208	0.91826748	Bertrand et al (2018)
Extant Rodentia	*Dipodomys ordii*	60.2	1.39	0.744137	1.21209601	Bertrand et al (2018)
Extant Rodentia	*Dipodomys panamintinus*	74	1.47	0.683542	1.09742492	Bertrand et al (2018)
Extant Rodentia	*Dipodomys spectabilis*	146.60	1.98	0.58396	0.89373706	Bertrand et al (2018)

Extant Rodentia	*Dolichotis patagonum*	5500	15	0.389823	0.46291125	Bertrand et al (2018)
Extant Rodentia	*Dolichotis patagonum*	5650	25.70	0.655963	0.77748522	Bertrand et al (2018)
Extant Rodentia	*Dolichotis patagonum*	5650	25.66	0.654942	0.77627512	Bertrand et al (2018)
Extant Rodentia	*Dolichotis patagonum*	7880	33.50	0.684212	0.79230113	Bertrand et al (2018)
Extant Rodentia	*Dolichotis patagonum*	7500	32	0.675581	0.78501781	Bertrand et al (2018)
Extant Rodentia	*Dolichotis patagonum*	5500	26	0.675693	0.8023795	Bertrand et al (2018)
Extant Rodentia	*Dolichotis patagonum*	7200	30	0.650919	0.75852531	Bertrand et al (2018)
Extant Rodentia	*Dremomys rufigenis*	418.43	5.59	0.815626	1.15993435	Bertrand et al (2018)
Extant Rodentia	*Eliurus minor*	38	1.46	1.063449	1.78890741	Bertrand et al (2018)
Extant Rodentia	*Eliurus myoxinus*	59	1.69	0.916716	1.49530947	Bertrand et al (2018)
Extant Rodentia	*Epixerus ebii*	605	9.77	1.114296	1.54430811	Bertrand et al (2018)
Extant Rodentia	*Erethizon dorsatum*	5160	27.90	0.756742	0.90264773	Bertrand et al (2018)
Extant Rodentia	*Erethizon dorsatum*	3430	28.70	1.023419	1.2561431	Bertrand et al (2018)
Extant Rodentia	*Erethizon dorsatum*	4980	25.10	0.697186	0.83367903	Bertrand et al (2018)
Extant Rodentia	*Erethizon dorsatum*	4980	27.20	0.755517	0.90342907	Bertrand et al (2018)
Extant Rodentia	*Erethizon dorsatum*	4640	27.10	0.789262	0.94846401	Bertrand et al (2018)
Extant Rodentia	*Erethizon dorsatum*	6200	27.10	0.649958	0.76537507	Bertrand et al (2018)
Extant Rodentia	*Erethizon dorsatum*	6640	24	0.549765	0.64429051	Bertrand et al (2018)
Extant Rodentia	*Erethizon dorsatum*	2800	30.77	1.257044	1.56496786	Bertrand et al (2018)
Extant Rodentia	*Erethizon dorsatum*	2725	21.22	0.882813	1.10115647	Bertrand et al (2018)

Extant Rodentia	*Erethizon dorsatum*	3410	19.15	0.685555	0.84179319	Bertrand et al (2018)
Extant Rodentia	*Erethizon dorsatum*	5000	24	0.664845	0.79478252	Bertrand et al (2018)
Extant Rodentia	*Eutamias sibiricus*	110	2.60	0.929093	1.45083368	Bertrand et al (2018)
Extant Rodentia	*Funisciurus anerythrus*	230	4.03	0.878238	1.30240885	Bertrand et al (2018)
Extant Rodentia	*Funisciurus carruthersi*	107.50	4	1.45156	2.27034886	Bertrand et al (2018)
Extant Rodentia	*Funisciurus carruthersi*	158	4.50	1.261627	1.92079554	Bertrand et al (2018)
Extant Rodentia	*Funisciurus carruthersi*	195.50	3.25	0.790012	1.1849762	Bertrand et al (2018)
Extant Rodentia	*Funisciurus carruthersi*	168	4.10	1.103177	1.67236033	Bertrand et al (2018)
Extant Rodentia	*Funisciurus carruthersi*	195	4.65	1.132266	1.69864337	Bertrand et al (2018)
Extant Rodentia	*Funisciurus isabella*	160.50	3.14	0.871917	1.32601361	Bertrand et al (2018)
Extant Rodentia	*Funisciurus lemniscatus*	154	3.22	0.918137	1.4003511	Bertrand et al (2018)
Extant Rodentia	*Funisciurus pyrropus*	258.75	4.47	0.899855	1.32350915	Bertrand et al (2018)
Extant Rodentia	*Funisciurus pyrropus*	301.15	4.34	0.789315	1.14866046	Bertrand et al (2018)
Extant Rodentia	*Funisciurus substriatus*	186.10	3.70	0.930784	1.40095071	Bertrand et al (2018)
Extant Rodentia	*Galea spixii*	672	6.20	0.658981	0.90659495	Bertrand et al (2018)
Extant Rodentia	*Geomys bursarius*	192.40	1.49	0.365037	0.54814874	Bertrand et al (2018)
Extant Rodentia	*Geomys pinetis*	313.50	2.30	0.408287	0.59249563	Bertrand et al (2018)
Extant Rodentia	*Gerbillus gerbillus*	95	3.40	1.340364	2.11464913	Bertrand et al (2018)
Extant Rodentia	*Gerbillus nanus*	18	0.52	0.62487	1.10758371	Bertrand et al (2018)
Extant Rodentia	*Gerbillus pyramidum*	79	1.14	0.508526	0.81271013	Bertrand et al (2018)

Extant Rodentia	*Gerbillus pyramidum*	70	1.20	0.580476	0.93558568	Bertrand et al (2018)
Extant Rodentia	*Gerbillus pyramidum*	72.30	1.08	0.511234	0.82212203	Bertrand et al (2018)
Extant Rodentia	*Gerbillus pyramidum*	79	1.06	0.472394	0.75496494	Bertrand et al (2018)
Extant Rodentia	*Gerbillus pyramidum*	57.90	1	0.551516	0.90079599	Bertrand et al (2018)
Extant Rodentia	*Gerbillus pyramidum*	59.40	0.98	0.529725	0.86365821	Bertrand et al (2018)
Extant Rodentia	*Glaucomys volans*	63.97	1.91	0.983817	1.59570287	Bertrand et al (2018)
Extant Rodentia	*Glaucomys volans*	52	1.89	1.115728	1.83609099	Bertrand et al (2018)
Extant Rodentia	*Glaucomys volans*	64	1.92	0.986233	1.59956877	Bertrand et al (2018)
Extant Rodentia	*Glis glis*	118	1.4	0.477294	0.74166916	Bertrand et al (2018)
Extant Rodentia	*Glis glis*	148	1.9	0.556541	0.85120624	Bertrand et al (2018)
Extant Rodentia	*Grammomys dolichurus*	45	1.18	0.767447	1.27579124	Bertrand et al (2018)
Extant Rodentia	*Graphiurus murinus*	17.70	0.55	0.66962	1.18830042	Bertrand et al (2018)
Extant Rodentia	*Heliosciurus gambianus*	209.80	3.53	0.819207	1.22270988	Bertrand et al (2018)
Extant Rodentia	*Heliosciurus rufobrachium*	354.98	5.79	0.943314	1.35705735	Bertrand et al (2018)
Extant Rodentia	*Heliosciurus rufobrachium isabellinus*	280.90	4.84	0.922494	1.3490281	Bertrand et al (2018)
Extant Rodentia	*Heliosciurus rufobrachium rufobrachium*	334	5.03	0.853794	1.23352259	Bertrand et al (2018)
Extant Rodentia	*Heterocephalus glaber*	39	0.52	0.372228	0.62501495	Bertrand et al (2018)
Extant Rodentia	*Heterogeomys cherriei*	405	3.19	0.476079	0.67859969	Bertrand et al (2018)
Extant Rodentia	*Heteromys desmarestianus*	77.10	1.08	0.487957	0.78116679	Bertrand et al (2018)

Extant Rodentia	*Heteromys pictus*	40.20	0.71	0.501025	0.83949778	Bertrand et al (2018)
Extant Rodentia	*Hoplomys gymnurus*	637	3.85	0.423875	0.585334	Bertrand et al (2018)
Extant Rodentia	*Hybomys univittatus*	56.80	1.15	0.63933	1.04562782	Bertrand et al (2018)
Extant Rodentia	*Hydrochoerus hydrochaeris*	31000	85	0.693466	0.72960221	Bertrand et al (2018)
Extant Rodentia	*Hydrochoerus hydrochaeris*	24130	61.40	0.59248	0.63438196	Bertrand et al (2018)
Extant Rodentia	*Hydrochoerus hydrochaeris*	29500	76	0.640991	0.67673739	Bertrand et al (2018)
Extant Rodentia	*Hydrochoerus hydrochaeris*	10000	55	0.957594	1.09052985	Bertrand et al (2018)
Extant Rodentia	*Hydrochoerus hydrochaeris*	12000	53	0.816664	0.91824086	Bertrand et al (2018)
Extant Rodentia	*Hydrochoerus hydrochaeris*	13000	53	0.774021	0.86543142	Bertrand et al (2018)
Extant Rodentia	*Hydrochoerus hydrochaeris*	17000	61	0.7443	0.8167187	Bertrand et al (2018)
Extant Rodentia	*Hydrochoerus hydrochaeris*	28000	52	0.454178	0.48126166	Bertrand et al (2018)
Extant Rodentia	*Hydrochoerus hydrochaeris*	24031	61.40	0.594114	0.63631487	Bertrand et al (2018)
Extant Rodentia	*Hydrochoerus hydrochaeris*	28500	75	0.647342	0.68509522	Bertrand et al (2018)
Extant Rodentia	*Hydrochoerus hydrochaeris*	27670	52.21	0.459649	0.48746314	Bertrand et al (2018)
Extant Rodentia	*Hydrochoerus hydrochaeris*	26700	75	0.676266	0.71898164	Bertrand et al (2018)
Extant Rodentia	*Hydrochoerus hydrochaeris*	34300	73	0.556538	0.58140676	Bertrand et al (2018)
Extant Rodentia	*Hydrochoerus hydrochaeris*	33000	75.50	0.590692	0.61875897	Bertrand et al (2018)
Extant Rodentia	*Hydrochoerus hydrochaeris*	54000	82	0.461242	0.46678548	Bertrand et al (2018)
Extant Rodentia	*Hydrochoerus hydrochaeris*	29500	76.02	0.641159	0.67691547	Bertrand et al (2018)
Extant Rodentia	*Hydrochoerus hydrochaeris*	28000	75	0.655065	0.6941274	Bertrand et al (2018)

Extant Rodentia	*Hydrochoerus hydrochaeris*	32500	71	0.561197	0.5884906	Bertrand et al (2018)
Extant Rodentia	*Hylopetes spadiceus*	84.22	2.02	0.863155	1.37330095	Bertrand et al (2018)
Extant Rodentia	*Hypogeomys antimena*	875	8	0.712465	0.96223001	Bertrand et al (2018)
Extant Rodentia	*Hystrix brachyura*	22000	43	0.441432	0.47571905	Bertrand et al (2018)
Extant Rodentia	*Hystrix cristata*	7036.50	36.50	0.804234	0.93869344	Bertrand et al (2018)
Extant Rodentia	*Hystrix cristata*	15000	32	0.424607	0.47002106	Bertrand et al (2018)
Extant Rodentia	*Hystrix cristata*	10000	37	0.6442	0.73362917	Bertrand et al (2018)
Extant Rodentia	*Hystrix* hybrid	13500	37	0.526862	0.5875294	Bertrand et al (2018)
Extant Rodentia	*Hystrix javanica*	23000	20	0.199293	0.21410473	Bertrand et al (2018)
Extant Rodentia	*Hystrix* sp.	15000	37.50	0.497587	0.55080593	Bertrand et al (2018)
Extant Rodentia	*Ictidomys tridecemlineatus*	200	2.20	0.526685	0.7887427	Bertrand et al (2018)
Extant Rodentia	*Ictidomys tridecemlineatus*	115	2.40	0.832458	1.29589468	Bertrand et al (2018)
Extant Rodentia	*Ictidomys tridecemlineatus*	139	2.16	0.660443	1.0145664	Bertrand et al (2018)
Extant Rodentia	*Ictidomys tridecemlineatus*	153	2.30	0.657511	1.00330033	Bertrand et al (2018)
Extant Rodentia	*Jaculus jaculus*	73	1.85	0.87009	1.39825918	Bertrand et al (2018)
Extant Rodentia	*Jaculus orientalis*	140	2.64	0.802633	1.23237832	Bertrand et al (2018)
Extant Rodentia	*Jaculus orientalis*	98	2.50	0.965244	1.51952406	Bertrand et al (2018)
Extant Rodentia	*Lagidium viscacia*	2460	12.40	0.552477	0.69407208	Bertrand et al (2018)
Extant Rodentia	*Lagidium viscacia*	2252	16	0.756341	0.95608003	Bertrand et al (2018)
Extant Rodentia	*Lagidium viscacia*	2350	15.20	0.698308	0.88009233	Bertrand et al (2018)

Extant Rodentia	*Lagidium viscacia*	3845	14.80	0.488877	0.59526903	Bertrand et al (2018)
Extant Rodentia	*Lagostomus maximus*	6630	20	0.458601	0.5375079	Bertrand et al (2018)
Extant Rodentia	*Lagostomus maximus*	6385	21	0.493833	0.5803301	Bertrand et al (2018)
Extant Rodentia	*Lagostomus maximus*	5965	17.20	0.423339	0.49986368	Bertrand et al (2018)
Extant Rodentia	*Lagostomus maximus*	3930	18	0.585933	0.71235567	Bertrand et al (2018)
Extant Rodentia	*Lagostomus maximus*	3765	16.50	0.552765	0.67405095	Bertrand et al (2018)
Extant Rodentia	*Lagostomus maximus*	2940	16.30	0.644486	0.79962332	Bertrand et al (2018)
Extant Rodentia	*Lagostomus maximus*	7000	17	0.375882	0.43888551	Bertrand et al (2018)
Extant Rodentia	*Lagostomus maximus*	1575	12.20	0.732826	0.94983263	Bertrand et al (2018)
Extant Rodentia	*Lagostomus maximus*	1990	14.50	0.744655	0.94949262	Bertrand et al (2018)
Extant Rodentia	*Lagostomus maximus*	1395	12	0.781871	1.02204778	Bertrand et al (2018)
Extant Rodentia	*Lagostomus maximus*	1500	11.10	0.688907	0.8959635	Bertrand et al (2018)
Extant Rodentia	*Lagostomus maximus*	1265	11.80	0.820919	1.08046297	Bertrand et al (2018)
Extant Rodentia	*Lagostomus maximus*	1125	13.20	0.993392	1.31824439	Bertrand et al (2018)
Extant Rodentia	*Lagostomus maximus*	1095	11.70	0.896597	1.19204948	Bertrand et al (2018)
Extant Rodentia	*Lagostomus maximus*	1440	13	0.8292	1.0815086	Bertrand et al (2018)
Extant Rodentia	*Lagostomus maximus*	1225	12.70	0.902758	1.19085226	Bertrand et al (2018)
Extant Rodentia	*Lagostomus maximus*	1510	12.60	0.778529	1.01205117	Bertrand et al (2018)
Extant Rodentia	*Lagostomus maximus*	1335	11.90	0.798534	1.04704583	Bertrand et al (2018)
Extant Rodentia	*Lagostomus maximus*	1325	11.90	0.802567	1.05288777	Bertrand et al (2018)

Extant Rodentia	*Lagostomus maximus*	1115	12.30	0.931214	1.236507	Bertrand et al (2018)
Extant Rodentia	*Lagostomus maximus*	1075	11	0.85343	1.13612284	Bertrand et al (2018)
Extant Rodentia	*Lagostomus maximus*	1030	11.20	0.894202	1.19396981	Bertrand et al (2018)
Extant Rodentia	*Lagostomus maximus*	1125	11.20	0.842878	1.1185104	Bertrand et al (2018)
Extant Rodentia	*Lagostomus maximus*	1230	11.70	0.829408	1.09378244	Bertrand et al (2018)
Extant Rodentia	*Lagostomus maximus*	3854	8.80	0.290229	0.35333192	Bertrand et al (2018)
Extant Rodentia	*Lagurus lagurus*	15	0.40	0.543122	0.97505099	Bertrand et al (2018)
Extant Rodentia	*Lariscus insignis*	324.71	4.65	0.803933	1.16378115	Bertrand et al (2018)
Extant Rodentia	*Lemmus lemmus*	21	0.70	0.75863	1.33024192	Bertrand et al (2018)
Extant Rodentia	*Lemmus trimucronatus*	32	1.13	0.920252	1.56675928	Bertrand et al (2018)
Extant Rodentia	*Lemmus trimucronatus*	48	1.31	0.817186	1.35235337	Bertrand et al (2018)
Extant Rodentia	*Lemniscomys striatus*	53.30	1.02	0.592259	0.9729643	Bertrand et al (2018)
Extant Rodentia	*Lemniscomys striatus*	53	1.01	0.586926	0.96458432	Bertrand et al (2018)
Extant Rodentia	*Lemniscomys striatus*	53.40	1.01	0.588036	0.96590028	Bertrand et al (2018)
Extant Rodentia	*Lemniscomys striatus*	57	0.99	0.549563	0.89859225	Bertrand et al (2018)
Extant Rodentia	*Lophuromys sikapusi*	63.50	1.20	0.619643	1.00554974	Bertrand et al (2018)
Extant Rodentia	*Lophuromys sikapusi*	70	1.13	0.546615	0.88100985	Bertrand et al (2018)
Extant Rodentia	*Macrotarsomys bastardi*	28.50	0.80	0.706582	1.21277339	Bertrand et al (2018)
Extant Rodentia	*Macrotarsomys ingens*	65	1.70	0.864203	1.40012853	Bertrand et al (2018)
Extant Rodentia	*Malacomys longipes*	98	1.29	0.496136	0.78103537	Bertrand et al (2018)

Extant Rodentia	*Malacothrix typicus*	124	0.50	0.164891	0.25533646	Bertrand et al (2018)
Extant Rodentia	*Marmota bobak*	5333	11.97	0.31761	0.37797396	Bertrand et al (2018)
Extant Rodentia	*Marmota marmota*	5000	16	0.44323	0.52985501	Bertrand et al (2018)
Extant Rodentia	*Marmota marmota*	4050	17	0.542341	0.65797149	Bertrand et al (2018)
Extant Rodentia	*Marmota marmota*	2950	17	0.670636	0.83187007	Bertrand et al (2018)
Extant Rodentia	*Marmota marmota*	3500	17	0.598056	0.73301597	Bertrand et al (2018)
Extant Rodentia	*Marmota sibirica*	1890	18.10	0.962206	1.2313222	Bertrand et al (2018)
Extant Rodentia	*Mastomys coucha*	21.80	0.71	0.753814	1.31834294	Bertrand et al (2018)
Extant Rodentia	*Mastomys natalensis*	63	0.90	0.4672	0.75858696	Bertrand et al (2018)
Extant Rodentia	*Meriones crassus*	122	1.36	0.453416	0.70292259	Bertrand et al (2018)
Extant Rodentia	*Meriones libycus*	93	1.51	0.603826	0.95405725	Bertrand et al (2018)
Extant Rodentia	*Meriones meridianus*	50	1.20	0.727261	1.20010242	Bertrand et al (2018)
Extant Rodentia	*Meriones shawi*	140	1.48	0.449961	0.69087875	Bertrand et al (2018)
Extant Rodentia	*Meriones unguiculatus*	50	1.30	0.787866	1.30011096	Bertrand et al (2018)
Extant Rodentia	*Mesocricetus auratus*	100	1.40	0.53327	0.83830664	Bertrand et al (2018)
Extant Rodentia	*Mesocricetus auratus*	125	1.12	0.367373	0.56856416	Bertrand et al (2018)
Extant Rodentia	*Mesocricetus auratus*	87	1.32	0.551969	0.87620263	Bertrand et al (2018)
Extant Rodentia	*Mesocricetus brandti*	80	1	0.442332	0.70629849	Bertrand et al (2018)
Extant Rodentia	*Microdipodops megacephalus*	13.60	0.48	0.690444	1.24806379	Bertrand et al (2018)
Extant Rodentia	*Microdipodops pallidus*	12.90	0.50	0.758246	1.37570417	Bertrand et al (2018)

Extant Rodentia	*Micromys minutus*	5.50	0.27	0.710047	1.36746979	Bertrand et al (2018)
Extant Rodentia	*Micromys minutus*	6.10	0.28	0.699676	1.33776522	Bertrand et al (2018)
Extant Rodentia	*Micromys minutus*	5.95	0.28	0.701354	1.34331267	Bertrand et al (2018)
Extant Rodentia	*Microtus agrestis*	42.50	0.90	0.608192	1.01510173	Bertrand et al (2018)
Extant Rodentia	*Microtus agrestris*	30.50	0.53	0.449003	0.76701635	Bertrand et al (2018)
Extant Rodentia	*Microtus arvalis arvalis*	20.50	0.40	0.440559	0.77381475	Bertrand et al (2018)
Extant Rodentia	*Microtus oeconomus stimmingi*	45	0.68	0.442258	0.73520173	Bertrand et al (2018)
Extant Rodentia	*Microtus pennsylvanicus*	23.70	0.66	0.660201	1.14788839	Bertrand et al (2018)
Extant Rodentia	*Microtus pennsylvanicus*	22.90	0.65	0.661044	1.15212065	Bertrand et al (2018)
Extant Rodentia	*Microtus pennsylvanicus*	25.20	0.72	0.687317	1.18991317	Bertrand et al (2018)
Extant Rodentia	*Microtus pennsylvanicus*	27.90	0.74	0.662435	1.13869445	Bertrand et al (2018)
Extant Rodentia	*Mus minutoides*	10.40	0.33	0.567485	1.04524464	Bertrand et al (2018)
Extant Rodentia	*Mus minutoides*	5	0.26	0.737025	1.42892822	Bertrand et al (2018)
Extant Rodentia	*Mus musculus*	18	0.43	0.516719	0.91588653	Bertrand et al (2018)
Extant Rodentia	*Mus musculus*	24	0.45	0.445954	0.77469555	Bertrand et al (2018)
Extant Rodentia	*Mus musculus*	20.85	0.43	0.468259	0.82149484	Bertrand et al (2018)
Extant Rodentia	*Mus musculus*	16	0.36	0.468124	0.83662065	Bertrand et al (2018)
Extant Rodentia	*Mus musculus*	24.32	0.48	0.46657	0.80975835	Bertrand et al (2018)
Extant Rodentia	*Mus musculus*	24	0.50	0.495504	0.86077284	Bertrand et al (2018)
Extant Rodentia	*Mus musculus*	18.92	0.42	0.492774	0.87040041	Bertrand et al (2018)

Extant Rodentia	*Mus musculus domesticus*	12	0.36	0.567636	1.03510224	Bertrand et al (2018)
Extant Rodentia	*Mus musculus musculus*	20	0.50	0.559885	0.98510535	Bertrand et al (2018)
Extant Rodentia	*Mus triton*	8.40	0.36	0.720865	1.34775421	Bertrand et al (2018)
Extant Rodentia	*Muscardinus avellanarius*	13	0.50	0.747217	1.35496099	Bertrand et al (2018)
Extant Rodentia	*Myocastor coypus*	3300	23	0.841671	1.03586316	Bertrand et al (2018)
Extant Rodentia	*Myocastor coypus*	3380	19	0.684224	0.84067883	Bertrand et al (2018)
Extant Rodentia	*Myocastor coypus*	5000	23	0.637143	0.76166658	Bertrand et al (2018)
Extant Rodentia	*Myocastor coypus*	5300	18.70	0.498191	0.59313333	Bertrand et al (2018)
Extant Rodentia	*Myocastor coypus*	3300	21	0.768482	0.94578811	Bertrand et al (2018)
Extant Rodentia	*Myocastor coypus*	7450	15.64	0.331674	0.38558131	Bertrand et al (2018)
Extant Rodentia	*Myocastor coypus*	7530	15.60	0.328466	0.38156733	Bertrand et al (2018)
Extant Rodentia	*Myocastor coypus*	8475	17.55	0.341383	0.39330422	Bertrand et al (2018)
Extant Rodentia	*Myocastor coypus*	5510	17.43	0.452423	0.53718029	Bertrand et al (2018)
Extant Rodentia	*Myocastor coypus*	3800	14.77	0.49175	0.59926029	Bertrand et al (2018)
Extant Rodentia	*Myocastor coypus*	5700	18.35	0.465606	0.55152289	Bertrand et al (2018)
Extant Rodentia	*Myocastor coypus*	5000	23	0.637143	0.76166658	Bertrand et al (2018)
Extant Rodentia	*Myocastor coypus f domestica*	3500	18.40	0.647308	0.79338199	Bertrand et al (2018)
Extant Rodentia	*Myodes glareolus*	35	0.593	0.456402	0.77218117	Bertrand et al (2018)
Extant Rodentia	*Myodes glareolus*	20	0.70	0.783839	1.37914749	Bertrand et al (2018)
Extant Rodentia	*Myoprocta pratti*	780	9.90	0.952249	1.29646279	Bertrand et al (2018)

Extant Rodentia	*Mystromys albicaudatus*	130	1.47	0.469671	0.72489336	Bertrand et al (2018)
Extant Rodentia	*Neotamias minimus*	37.05	1.45	1.073591	1.80917233	Bertrand et al (2018)
Extant Rodentia	*Oenomys hypoxanthus*	92	1.15	0.463211	0.73243606	Bertrand et al (2018)
Extant Rodentia	*Oenomys hypoxanthus*	92	1.17	0.469656	0.74262647	Bertrand et al (2018)
Extant Rodentia	*Oenomys hypoxanthus*	92	1.16	0.468044	0.74007887	Bertrand et al (2018)
Extant Rodentia	*Oenomys hypoxanthus*	178	1.48	0.383088	0.57839667	Bertrand et al (2018)
Extant Rodentia	*Ondatra zibethicus*	1500	4.80	0.297906	0.38744367	Bertrand et al (2018)
Extant Rodentia	*Ondatra zibethicus*	1500	7.60	0.471684	0.61345248	Bertrand et al (2018)
Extant Rodentia	*Ondatra zibethicus*	1600	8.10	0.481441	0.62332006	Bertrand et al (2018)
Extant Rodentia	*Ondatra zibethicus*	1032	5.70	0.454494	0.6067737	Bertrand et al (2018)
Extant Rodentia	*Ondatra zibethicus*	900	5.33	0.465804	0.62785972	Bertrand et al (2018)
Extant Rodentia	*Ondatra zibethicus*	900	5.33	0.465804	0.62785972	Bertrand et al (2018)
Extant Rodentia	*Orthogeomys heterodus*	630	3.96	0.439708	0.60766874	Bertrand et al (2018)
Extant Rodentia	*Orthogeomys hispidus*	542.10	3.66	0.448877	0.62689966	Bertrand et al (2018)
Extant Rodentia	*Otomys irroratus*	57.70	0.97	0.534862	0.87380724	Bertrand et al (2018)
Extant Rodentia	*Otomys irroratus*	66	1.62	0.815153	1.31925081	Bertrand et al (2018)
Extant Rodentia	*Paraxerus cepapi*	138.13	2.91	0.892084	1.37101409	Bertrand et al (2018)
Extant Rodentia	*Paraxerus cepapi*	223.80	3.22	0.714727	1.06195425	Bertrand et al (2018)
Extant Rodentia	*Paraxerus poensis*	100	2.73	1.041145	1.63669391	Bertrand et al (2018)
Extant Rodentia	*Pelomys campanae*	91	1.23	0.499075	0.78974948	Bertrand et al (2018)

Extant Rodentia	*Pelomys fallax*	121	1.46	0.489447	0.7592181	Bertrand et al (2018)
Extant Rodentia	*Pelomys fallax*	110	1.41	0.502425	0.78456621	Bertrand et al (2018)
Extant Rodentia	*Pelomys fallax*	111	1.41	0.501519	0.78265536	Bertrand et al (2018)
Extant Rodentia	*Perognathus flavus*	8	0.29	0.591126	1.10897111	Bertrand et al (2018)
Extant Rodentia	*Perognathus longimembris*	8	0.30	0.610831	1.14593682	Bertrand et al (2018)
Extant Rodentia	*Perognathus parvus*	17.3	0.45	0.552378	0.98181421	Bertrand et al (2018)
Extant Rodentia	*Perognathus parvus*	17	0.58	0.724179	1.28875588	Bertrand et al (2018)
Extant Rodentia	*Peromyscus californicus*	55	1.03	0.585161	0.95919356	Bertrand et al (2018)
Extant Rodentia	*Peromyscus californicus*	51.20	1.03	0.614093	1.011676	Bertrand et al (2018)
Extant Rodentia	*Peromyscus eremicus*	20.10	0.65	0.726092	1.27709688	Bertrand et al (2018)
Extant Rodentia	*Peromyscus eremicus*	20	0.64	0.718444	1.26408718	Bertrand et al (2018)
Extant Rodentia	*Peromyscus gossypinus*	27.20	0.68	0.619679	1.06709428	Bertrand et al (2018)
Extant Rodentia	*Peromyscus leucopus*	25.50	0.63	0.599484	1.03699267	Bertrand et al (2018)
Extant Rodentia	*Peromyscus leucopus*	19.70	0.64	0.720779	1.26953778	Bertrand et al (2018)
Extant Rodentia	*Peromyscus leucopus*	22.90	0.62	0.636193	1.10880918	Bertrand et al (2018)
Extant Rodentia	*Peromyscus maniculatus bairdii*	16.50	0.52	0.660088	1.17715637	Bertrand et al (2018)
Extant Rodentia	*Peromyscus maniculatus bairdii*	18	0.52	0.623428	1.10502774	Bertrand et al (2018)
Extant Rodentia	*Peromyscus maniculatus gracilis*	22.10	0.69	0.721074	1.25987811	Bertrand et al (2018)
Extant Rodentia	*Peromyscus maniculatus gracilis*	26	0.69	0.648936	1.12101077	Bertrand et al (2018)
Extant Rodentia	*Peromyscus polionotus*	13.50	0.49	0.714426	1.2920811	Bertrand et al (2018)

Extant Rodentia	*Peromyscus polionotus*	13.10	0.49	0.723616	1.31146123	Bertrand et al (2018)
Extant Rodentia	*Petaurista petaurista*	1096.65	11.73	0.898077	1.19389145	Bertrand et al (2018)
Extant Rodentia	*Petinomys setosus*	41.86	1.44	0.983395	1.64307785	Bertrand et al (2018)
Extant Rodentia	*Podomys floridanus*	36.50	0.89	0.665698	1.12298233	Bertrand et al (2018)
Extant Rodentia	*Podomys floridanus*	39.20	0.89	0.636115	1.06773123	Bertrand et al (2018)
Extant Rodentia	*Praomys jacksoni*	49	0.87	0.534449	0.88317959	Bertrand et al (2018)
Extant Rodentia	*Protoxerus aubinni*	525	8.03	1.006816	1.40927342	Bertrand et al (2018)
Extant Rodentia	*Protoxerus stangeri*	767.23	9.28	0.90281	1.23057362	Bertrand et al (2018)
Extant Rodentia	*Protoxerus stangeri*	690.60	10.25	1.069449	1.46848724	Bertrand et al (2018)
Extant Rodentia	*Pteromys buechneri*	106.37	2.22	0.81127	1.26982654	Bertrand et al (2018)
Extant Rodentia	*Pteromys nitidus*	1600	11.80	0.701359	0.9080465	Bertrand et al (2018)
Extant Rodentia	*Pteromyscus pulverulentus*	195.44	3.45	0.838463	1.25767647	Bertrand et al (2018)
Extant Rodentia	*Rattus norvegicus*	274	2.18	0.422651	0.6191501	Bertrand et al (2018)
Extant Rodentia	*Rattus norvegicus*	278	2.30	0.441608	0.64626339	Bertrand et al (2018)
Extant Rodentia	*Rattus norvegicus*	197	1.61	0.389361	0.58370812	Bertrand et al (2018)
Extant Rodentia	*Rattus norvegicus*	291	2.27	0.422704	0.61662328	Bertrand et al (2018)
Extant Rodentia	*Rattus norvegicus*	305	2.36	0.425843	0.61916299	Bertrand et al (2018)
Extant Rodentia	*Rattus norvegicus*	448	2.36	0.329135	0.46584468	Bertrand et al (2018)
Extant Rodentia	*Rattus norvegicus domestica*	324	2.70	0.467862	0.67738461	Bertrand et al (2018)
Extant Rodentia	*Rattus norvegicus norvegicus*	202	2.60	0.618311	0.92531202	Bertrand et al (2018)

Extant Rodentia	*Rattus rattus*	160	2.24	0.622739	0.94727069	Bertrand et al (2018)
Extant Rodentia	*Rattus rattus*	200	1.59	0.38065	0.57004586	Bertrand et al (2018)
Extant Rodentia	*Rattus rattus*	150	1.92	0.557364	0.85166454	Bertrand et al (2018)
Extant Rodentia	*Rattus rattus alexandrinus*	150	1.57	0.455761	0.69641319	Bertrand et al (2018)
Extant Rodentia	*Rattus rattus rattus*	217	1.68	0.380803	0.56702727	Bertrand et al (2018)
Extant Rodentia	*Ratufa affinis*	1074.27	11.73	0.910274	1.21185368	Bertrand et al (2018)
Extant Rodentia	*Ratufa bicolor*	1440	12	0.765415	0.99831563	Bertrand et al (2018)
Extant Rodentia	*Ratufa bicolor*	1400	12	0.779999	1.0193454	Bertrand et al (2018)
Extant Rodentia	*Ratufa indica*	1935	11.40	0.596551	0.76214184	Bertrand et al (2018)
Extant Rodentia	*Ratufa indica*	1010	11.60	0.938386	1.25468597	Bertrand et al (2018)
Extant Rodentia	*Rhinosciurus laticaudatus*	507.38	4.17	0.535657	0.75157065	Bertrand et al (2018)
Extant Rodentia	*Sciurus carolinensis*	592.55	7.67	0.886828	1.23084865	Bertrand et al (2018)
Extant Rodentia	*Sciurus carolinesis*	469	7.58	1.025185	1.44635923	Bertrand et al (2018)
Extant Rodentia	*Sciurus carolinesis*	466	7.48	1.016019	1.43407177	Bertrand et al (2018)
Extant Rodentia	*Sciurus carolinesis*	535	7.53	0.932431	1.303431	Bertrand et al (2018)
Extant Rodentia	*Sciurus granatensis*	336.99	6.02	1.01647	1.46763308	Bertrand et al (2018)
Extant Rodentia	*Sciurus niger*	365	6.90	1.103905	1.5849926	Bertrand et al (2018)
Extant Rodentia	*Sciurus niger*	365	6.50	1.03991	1.49310897	Bertrand et al (2018)
Extant Rodentia	*Sciurus niger*	703	10.20	1.051862	1.44254078	Bertrand et al (2018)
Extant Rodentia	*Sciurus niger*	770	10.50	1.01873	1.38822874	Bertrand et al (2018)

Extant Rodentia	*Sciurus niger*	650	9.20	0.999896	1.37881758	Bertrand et al (2018)
Extant Rodentia	*Sciurus niger*	580	8.95	1.049893	1.4593558	Bertrand et al (2018)
Extant Rodentia	*Sciurus niger cinereus*	328	7.20	1.237416	1.79003169	Bertrand et al (2018)
Extant Rodentia	*Sciurus vulgaris*	350	7.50	1.234112	1.77715693	Bertrand et al (2018)
Extant Rodentia	*Sciurus vulgaris*	323	6.10	1.059212	1.53389219	Bertrand et al (2018)
Extant Rodentia	*Sciurus vulgaris*	287	5.81	1.091978	1.59447751	Bertrand et al (2018)
Extant Rodentia	*Sciurus vulgaris*	316	5.89	1.037872	1.50529536	Bertrand et al (2018)
Extant Rodentia	*Sciurus vulgaris*	327	6.23	1.072901	1.55237835	Bertrand et al (2018)
Extant Rodentia	*Sciurus vulgaris*	361	5.85	0.942855	1.35480068	Bertrand et al (2018)
Extant Rodentia	*Sciurus vulgaris*	389	6	0.919822	1.31481126	Bertrand et al (2018)
Extant Rodentia	*Sigmodon hispidus*	148	1.18	0.345641	0.52864388	Bertrand et al (2018)
Extant Rodentia	*Spalax leucodon*	122	3	1.000182	1.55056454	Bertrand et al (2018)
Extant Rodentia	*Spalax leucodon*	180	2.63	0.675681	1.01936323	Bertrand et al (2018)
Extant Rodentia	*Spalax leucodon*	214	1.90	0.434705	0.64792133	Bertrand et al (2018)
Extant Rodentia	*Spermophilus citellus*	290	2.58	0.481539	0.70261927	Bertrand et al (2018)
Extant Rodentia	*Spermophilus suslicus*	224	2.30	0.510364	0.75826179	Bertrand et al (2018)
Extant Rodentia	*Stochomys longicaudatus*	65	1.27	0.64561	1.04597837	Bertrand et al (2018)
Extant Rodentia	*Tachyoryctes splendens*	282	2.10	0.399366	0.58386148	Bertrand et al (2018)
Extant Rodentia	*Tachyoryctes splendens*	174	2.29	0.601847	0.910131	Bertrand et al (2018)
Extant Rodentia	*Tachyoryctes splendens*	206	2.40	0.563299	0.84182994	Bertrand et al (2018)

Extant Rodentia	*Tachyoryctes splendens*	218	2.24	0.506175	0.75346847	Bertrand et al (2018)
Extant Rodentia	*Tamias striatus*	80	2.70	1.194297	1.90700593	Bertrand et al (2018)
Extant Rodentia	*Tamias striatus*	97	2.90	1.127404	1.77607697	Bertrand et al (2018)
Extant Rodentia	*Tamias striatus*	75	2.22	1.02537	1.64468415	Bertrand et al (2018)
Extant Rodentia	*Tamias striatus*	62	2.31	1.212072	1.97023032	Bertrand et al (2018)
Extant Rodentia	*Tamiasciurus hudsonicus*	256.61	4.90	0.99299	1.46134237	Bertrand et al (2018)
Extant Rodentia	*Tamiasciurus hudsonicus*	125	4.80	1.574455	2.43670355	Bertrand et al (2018)
Extant Rodentia	*Tamiasciurus hudsonicus*	159	4.10	1.144634	1.74190655	Bertrand et al (2018)
Extant Rodentia	*Tamiasciurus hudsonicus*	248	5.02	1.040495	1.53491504	Bertrand et al (2018)
Extant Rodentia	*Tamiasciurus hudsonicus*	183	4.71	1.196733	1.80335808	Bertrand et al (2018)
Extant Rodentia	*Tamiasciurus hudsonicus*	169	4.21	1.12828	1.70970351	Bertrand et al (2018)
Extant Rodentia	*Tamiasciurus hudsonicus*	183	3.97	1.008712	1.52002793	Bertrand et al (2018)
Extant Rodentia	*Tamiasciurus hudsonicus*	159	4.80	1.340059	2.03930523	Bertrand et al (2018)
Extant Rodentia	*Thamnomys venustus*	82.50	1.29	0.559398	0.89130263	Bertrand et al (2018)
Extant Rodentia	*Thamnomys venustus*	82.50	1.28	0.556365	0.88646985	Bertrand et al (2018)
Extant Rodentia	*Thamnomys venustus*	82.50	1.35	0.584963	0.93203606	Bertrand et al (2018)
Extant Rodentia	*Thomomys bottae*	185.20	1.41	0.355281	0.53492508	Bertrand et al (2018)
Extant Rodentia	*Thomomys talpoides*	90.60	1.01	0.410827	0.65030421	Bertrand et al (2018)
Extant Rodentia	*Thryonomys gregorianus*	3500	13.15	0.462614	0.56700941	Bertrand et al (2018)
Extant Rodentia	*Thryonomys gregorianus*	3500	13.15	0.462614	0.56700941	Bertrand et al (2018)

Extant Rodentia	*Thryonomys swinderianus*	4500	12	0.356737	0.42961442	Bertrand et al (2018)
Extant Rodentia	*Urocitellus columbianus*	482	3.52	0.467939	0.65891969	Bertrand et al (2018)
Extant Rodentia	*Urocitellus columbianus*	529	3.57	0.4456	0.62338973	Bertrand et al (2018)
Extant Rodentia	*Urocitellus parryii*	878	5.63	0.500249	0.6754564	Bertrand et al (2018)
Extant Rodentia	*Urocitellus parryii*	958	5.74	0.481078	0.64561897	Bertrand et al (2018)
Extant Rodentia	*Urocitellus parryii*	482	3.95	0.524851	0.73905857	Bertrand et al (2018)
Extant Rodentia	*Urocitellus parryii*	756	4.98	0.48924	0.6675459	Bertrand et al (2018)
Extant Rodentia	*Urocitellus richardsonii*	354	3.08	0.502342	0.72281254	Bertrand et al (2018)
Extant Rodentia	*Urocitellus richardsonii*	361	3.31	0.534169	0.76755496	Bertrand et al (2018)
Extant Rodentia	*Xerus inauris*	638	6.41	0.705366	0.97394253	Bertrand et al (2018)
Extant Rodentia	*Zapus hudsonius*	19.30	0.70	0.798302	1.40810193	Bertrand et al (2018)
Extant Rodentia	*Zapus hudsonius*	15.20	0.71	0.948796	1.70176556	Bertrand et al (2018)
Extant Rodentia	*Zygogeomys trichopus*	545	3.63	0.443781	0.6195511	Bertrand et al (2018)

Table S6. Data for bivariate plot (Fig. 4)

Group	Species	Specimen number	Total Endocranial Volume (mm3)	Log Endocranial Volume	Body mass (g)	Log Body Mass	Source
Extant Leporidae	*Brachylagus idahoensis*	AMNH 92869	5145.19	3.711401417	339.56	2.530917909	This paper
Extant Leporidae	*Poelagus marjorita*	AMNH 51052	11807.96	4.072174918	2480.24	3.394493972	This paper
Extant Leporidae	*Lepus americanus bairdii*	AMNH 99352	9538.09	3.979461416	1396.22	3.144953375	This paper
Extant Leporidae	*Lepus americanus phaeonotus*	AMNH 97648	10221.40	4.009510384	998.68	2.999426961	This paper
Extant Leporidae	*Lepus arcticus*	AMNH 42139	15949.90	4.202757965	4003.10	3.602396965	This paper
Extant Leporidae	*Oryctolagus cuniculus*	AMNH 34816	9363.12	3.971420589	1796.07	3.254323205	This paper
Extant Leporidae	*Romerolagus diazi*	AMNH 148172	6020.62	3.779641217	1027.79	3.011904802	This paper
Extant Ochotonidae	*Ochotona pallasi*	AMNH 59712	2138.24	3.33005645	223.85	2.349951356	This paper
Extant Ochotonidae	*Ochotona princeps princeps*	AMNH 120698	2270.48	3.356117681	202.92	2.307332896	This paper
Extant Ochotonidae	*Ochotona princeps schisticeps*	AMNH 40547	2479.85	3.394425412	155.10	2.190612212	This paper
Stem lagomorph	*Megalagus turgidus*	UC 1642	7052.78	3.848360337	2325.01	3.366424825	This paper
Extinct Rodentia	*Prosciurus relictus*	USNM 437793	957.45	2.981113836	30.07	1.478133428	Bertrand et al (2018)

Extinct Rodentia	*Protosciurus* cf. *rachelae*	YPM 14736	4546.82	3.657707762	349.62	2.543596268	Bertrand et al (2018)
Extinct Rodentia	*Paramys copei*	AMNH 4756	7526.65	3.876601721	1029.89	3.012790841	Bertrand et al (2018)
Extinct Rodentia	*Paramys delicatus*	AMNH 12506	12565.40	4.099176318	2913.82	3.46446272	Bertrand et al (2018)
Extinct Rodentia	*Pseudotomus horribilis*	USNM 17159	15188.20	4.181506307	7466.70	3.873128748	Bertrand et al (2018)
Extinct Rodentia	*Pseudotomus oweni*	USNM 17161	12063.00	4.081455328	5396.00	3.732071693	Bertrand et al (2018)
Extinct Rodentia	*Pseudotomus petersoni*	AMNH 2018	17014.90	4.230829401	6644.56	3.822466227	Bertrand et al (2018)
Extinct Rodentia	*Pseudotomus hians*	AMNH 5025	13679.10	4.136057524	3153.50	3.498792835	Bertrand et al (2018)
Extinct Rodentia	*Rapamys atramontis*	AMNH 128706	7109.97	3.851867768	1307.61	3.116477912	Bertrand et al (2018)
Extinct Rodentia	*Rapamys atramontis*	AMNH 128704	6006.47	3.778619312	918.93	2.96328243	Bertrand et al (2018)
Extinct Rodentia	*Ischyromys typus*	ROMV 1007	5578.07	3.74648396	1342.23	3.127826941	Bertrand et al (2018)
Extinct Rodentia	*Ischyromys typus*	AMNH 12252	5934.55	3.773387793	1086.42	3.035997752	Bertrand et al (2018)
Extinct Rodentia	*Ischyromys typus*	AMNH F:AM 144638	7276.91	3.861947004	1109.01	3.044935462	Bertrand et al (2018)
Extinct Rodentia	*Cedromus wilsoni*	USNM 256584	3609.87	3.557491562	268.89	2.429574651	Bertrand et al (2018)
Plesiadapiformes	*Microsyops annectens*	UW 12362	5900	3.770852012	1686	3.22685757	Silcox et al (2010a)
Plesiadapiformes	*Ignacius graybullianus*	USNM 421608	2140	3.330413773	253	2.403120521	Silcox et al (2009)
Plesiadapiformes	*Plesiadapis tricuspidens*	MNHN CR 125	5210	3.716837723	6372	3.804275767	Orliac et al (2014)
Plesiadapiformes	*Plesiadapis cookei*	UM 87990	5000	3.698970004	2200	3.342422681	Gingerich & Gunnell (2005)
Apatemyidae	*Labidolemur kayi*	USNM 530208/530221	501.88	2.700596428	74	1.86923172	Silcox et al (2011)

Extinct Euprimates	*Notharctus tenebrosus*	AMNH 127167	7380	3.868056362	2641	3.421768401	Harrington et al (2016)
Extinct Euprimates	*Notharctus tenebrosus*	USNM V 23277	8060	3.906335042	2923	3.465828815	Harrington et al (2016)
Extinct Euprimates	*Notharctus tenebrosus*	USNM V 23278	7430	3.870988814	2244	3.351022853	Harrington et al (2016)
Extinct Euprimates	*Smilodectes gracilis*	USNM V 17994	8630	3.936010796	1420	3.152288344	Harrington et al (2016)
Extinct Euprimates	*Smilodectes gracilis*	USNM V 17996	8990	3.953759692	1303	3.114944416	Harrington et al (2016)
Extinct Euprimates	*Smilodectes gracilis*	USNM V 21815	7440	3.871572936	1582	3.199206479	Harrington et al (2016)
Extinct Euprimates	*Smilodectes gracilis*	UM 32773	8420	3.925312091	1547	3.189490314	Harrington et al (2016)
Extinct Euprimates	*Adapis parisiensis*	NHM M 1345	8810	3.944975908	1074	3.031004281	Harrington et al (2016)
Extinct Euprimates	*Rooneyia viejaensis*	TMM 40688-7	7234	3.859378504	381	2.580924976	Kirk et al (2014)
Extinct Euprimates	*Microchoerus erinaceus*	UM-PRR 1771	4260	3.629409599	597	2.775974331	Ramdarshan & Orliac (2016)

Table S7. Metrical data of the width of the occipital condyles (in mm) and body mass estimation (in g; using formula of Moncunill-Solé et al. 2015) for studied lagomorph taxa

Family	Species	Specimen number	Occipital width	Body mass
Leporidae	*Brachylagus idahoensis*	AMNH 92869	9.81	339.5
	Poelagus marjorita	AMNH 51052	15.95	2480.2
	Lepus americanus phaeonotus	AMNH 97648	12.77	998.6
	Lepus americanus bairdii	AMNH 99352	13.86	1396.2
	Lepus arcticus	AMNH 42139	17.93	4003.1
	Oryctolagus cuniculus	AMNH 34816	14.74	1796.1
	Romerolagus diazi	AMNH 148172	12.86	1027.8
Ochotonidae	*Ochotona princeps princeps*	AMNH 120698	8.65	202.9
	Ochotona princeps schisticep	AMNH 40547	8.10	155.1
	Ochotona pallasi	AMNH 59712	8.86	223.8
Stem Lagomorpha	*Megalagus turgidus*	UC 1642	15.70	2325.0
